# Biphasic Effects on Allergen‐Specific Type 2 Memory B Cells Over 18 Months Sublingual Immunotherapy for House Dust Mite Allergy

**DOI:** 10.1111/all.70342

**Published:** 2026-04-17

**Authors:** Lin Hsin, Simone Reinwald, Anouk von Borstel, Pei Mun Aui, Kirsten Deckert, P. Mark Hogarth, Laurent Mascarell, Mark Hew, Robyn E. O'Hehir, Menno C. van Zelm

**Affiliations:** ^1^ Department of Immunology, School of Translational Medicine Monash University Melbourne Victoria Australia; ^2^ Allergy, Asthma and Clinical Immunology, Alfred Health Melbourne Victoria Australia; ^3^ Department of Immunology Leiden University Medical Center Leiden the Netherlands; ^4^ Immune Therapies Group Burnet Institute Melbourne Victoria Australia; ^5^ Department of Clinical Pathology the University of Melbourne Parkville Victoria Australia; ^6^ Innovation & Science Department Stallergenes Greer Antony France; ^7^ Public Health & Preventive Medicine Monash University Melbourne Victoria Australia; ^8^ Department of Immunology, Erasmus MC University Medical Center Rotterdam the Netherlands

**Keywords:** allergen immunotherapy, allergic rhinitis (AR), atopic asthma, house dust mite, type 2 memory B cells

## Abstract

**Background:**

Type 2 memory B cells (Bmem) are the reservoir of pathogenic IgE in allergies. Allergen immunotherapy (AIT) can change the course of disease, but if it involves reprogramming of allergen‐reactive Bmem remains unknown. Here, we examine how AIT affects allergen‐specific Bmem in house dust mite (HDM) allergic patients.

**Methods:**

HDM allergic patients were longitudinally evaluated over 18 months with or without sublingual HDM‐AIT. Visual analog scores, lung function tests, medication scores, serum IgE, and flowcytometric analysis of Der p 1 and Der p 2‐specific Type 2 Bmem were performed at t = 0, 4, 12, and 18 months.

**Results:**

Patients on HDM‐AIT showed clinical improvement over 18 months with reduced intake of other medications and increases in specific serum IgE, IgG2, and IgG4. Allergen‐specific Bmem and the proportions of Type 2 Bmem therein became more abundant at 4 and 12 months and showed upregulation of CD29 and IgG4. At 18 months, the Type 2 Bmem proportions were reduced.

**Conclusion:**

A biphasic Bmem response with early phenotypic changes followed by loss of the Type 2 state was observed during 18 months of AIT. As durable unresponsiveness takes 18 months of AIT, deletion of Type 2 Bmem is likely an important step in disease attenuation. Interventions that expedite this outcome may be beneficial in driving remission.

## Graphical abstract

Rapid clinical relief of allergy symptoms during 18 months of sublingual immunotherapy (SLIT) for house dust mite (HDM) is associated with increased serum IgE, IgG2, and IgG4 to Der p 1 and Der p 2. Der p 1 and Der p 2 specific Type 2 memory B cells are expanded at 4 months and 12 months of SLIT, with increased expression of CD29 and IgG4. Following their early expansion, allergen‐specific type 2 Bmem populations declined at 18 months, paralleling the decline in serum‐specific IgE. AIT, allergen immunotherapy; Bmem, memory B cell; Der p, *Dermatophagoides pteronyssinus*; HDM, house dust mite; Ig, immunoglobulin; SLIT, sublingual immunotherapy.

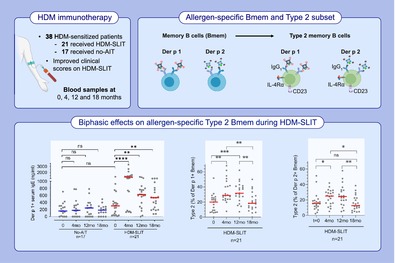

## 1 Introduction

Allergen immunotherapy (AIT) is the only disease‐modifying treatment for IgE‐mediated respiratory allergic diseases, such as allergic rhinitis (AR) and atopic asthma, which are prevalent and have significant global health and economic impacts [1]. AIT involves regular administration of standardized doses of allergen in the form of unfractionated allergen extract or purified components [2]. AIT ameliorates symptoms early on [3, 4], and following 18 months, sustained clinical tolerance upon discontinuation can be observed [5, 6]. However, the immunological mechanisms driving the early‐stage and long‐term clinical efficacy are not yet fully understood.

House dust mite (HDM) is the most prevalent perennial allergen among AR and asthma patients globally [7, 8]. HDM (*Dermatophagoides pteronyssinus*) contains multiple allergen components, with 95% of HDM‐allergic patients sensitized to group 1 (Der p 1) and/or 2 (Der p 2) [8, 9]. HDM‐AIT was found effective for treatment with duration varying from two to five years in European and Asian patient cohorts [10, 11, 12]. However, HDM‐AIT is not effective in all patients [3], highlighting the need to understand factors predicting and enhancing treatment responsiveness.

Successful AIT, including sublingual AIT (SLIT) for HDM allergy, has been shown to shift Th2 dominance to tolerogenic allergen‐specific Treg and Th1 [13, 14], and to modify antibody responses by increasing serum allergen‐specific IgG4, IgG2, and IgA [15, 16]. In particular, IgG4 is thought to competitively inhibit IgE‐mediated activation of effector cells, i.e., mast cells and basophils [17, 18], and IgG2 to inhibit basophil activation via engaging the inhibitory FcγRIIb [19]. High‐responders to one year of HDM‐SLIT show increased HDM‐specific serum IgG2, which correlates with clinical benefit as read out by reduced symptom and medication scores [20].

The serological changes on AIT are mirrored in the allergen‐specific memory B cell (Bmem) compartment, the reservoir for allergen‐specific antibody responses. Early increases in IgG2 and IgG4‐expressing allergen‐specific Bmem are seen for both ryegrass pollen (RGP) SLIT (4 months) and bee venom ultra‐rush AIT (2 months), correlating with clinical efficacy [14, 21]. Furthermore, AIT induces transcriptional and immunophenotypic changes in the allergen‐specific Bmem [22], notably within the recently‐defined ‘Type 2’ Bmem subset, a subset defined as expressing germline IgE transcripts, surface CD23 and IL4Rα, and showing an increased propensity to differentiate into IgE antibody‐producing cells in vitro [23, 24]. With the propensity of Type 2 Bmem to undergo Ig class‐switching to IgE in peanut, grass pollen, and bee venom‐allergic individuals [21, 23, 24, 25], these are now considered the reservoir of IgE in protracted allergic disease [26]. Paradoxically, this population expands following clinically successful AIT, displaying transcriptional shifts, including the upregulation of *ITGB1*, encoding Integrin β1 (CD29), and *IGHG4* [22, 25]. Enhanced expression of CD29 may dampen the allergen‐specific B cell response [27], resulting in rapid alleviation of disease within two to four months of treatment [28, 29]. However, it remains unclear whether the upregulation of CD29 and dominant use of IgG4 among type 2 Bmem is sufficient to explain disease remission, or if additional long‐term changes are required for AIT‐induced sustained clinical tolerance [26].

Here, we assessed the Bmem compartment over 18 months in 38 HDM‐allergic individuals with or without HDM‐SLIT administration. Using recombinant Der p 1 and Der p 2 tetramers [30], we investigated allergen‐specific Bmem, particularly the type 2 phenotype, to identify the immunological changes induced by clinically effective AIT.

## 2 Methods

### 2.1 Study Design and Participants

HDM‐allergic patients were recruited from the Allergy Clinic at the Alfred Hospital in Melbourne, Victoria, Australia. Patients with AR with or without atopic asthma were recruited following a diagnosis of perennial allergy with confirmation of HDM sensitization (serum HDM‐specific IgE ≥ 0.35 kUA/L; ImmunoCAP, Phadia, Uppsala, Sweden), and based on stable lung function (FEV1 ≥ 70% predicted). All patients were permitted to use standard pharmacotherapy, including non‐sedating antihistamines and topical intranasal corticosteroids. Patients in the treatment group received HDM (Der p and Der f extract) sublingual allergen immunotherapy (HDM‐SLIT; *n* = 21) over 18 months. Treatment with SLIT (Actair; Stallergenes Greer, Antony, France) required daily dosing by dissolving a tablet under the tongue for two to three minutes before swallowing, and the regimen involved day 1–1 tablet 100 index of reactivity (IR); day 2–2 tablets of 100IR; and day 3 and subsequent days—1 tablet of 300IR. Patients in the no‐AIT arm (*n* = 17) were evaluated at the same time points as HDM‐SLIT patients (0, 4, 12, 18 months). Study exclusion criteria included pregnancy or administration of AIT within the past five years, and current treatment with beta‐blockers or oral corticosteroids. No subjects experienced anaphylaxis in response to SLIT. All subjects were recruited according to the principles of the Declaration of Helsinki and provided with written consent before inclusion (Alfred Project ID: 48/21).

### 2.2 Clinical Evaluation and Blood Sample Collection

Following recruitment, each patient was asked to complete a visual analog score (VAS), symptom diary score, and medication score 2 weeks prior to each study visit (t = 0, 4 months, 12 months, and 18 months) [31, 32]. VAS was measured based on the experience of the severity of AR symptoms. Participants were asked to rate their symptom severity by marking a point on a 100 mm horizontal line, with 0 mm as no symptoms and 100 mm as “extremely severe symptoms”, and the VAS was then quantified via the distance from 0 mm to the indicated point by participants. Symptom severity was rated for multiple affected locations (i.e., chest, nose, eye and throat) on a scale of 0–3, where 0 indicated the absence of symptoms; 1 indicated mild symptoms that are present but not bothersome and being easily tolerated; 2 indicated moderate symptoms that are bothersome but tolerable and not disabling; and 3 denoted severe symptoms that are difficult to tolerate, disabling, and interfere with daily activities, sleep, or both. The medication score represents the total number of medications used over the two weeks, including nasal sprays, antihistamines, reliever inhalers, preventer inhalers, and other treatments (e.g., eye drops). The score was calculated based on the type and frequency of medication use. For example, if a reliever inhaler was used twice daily at the standard dose (4 puffs), this resulted in a score of 2 per day, leading to a total score of 28 over two weeks. Fractional exhaled nitric oxide (FeNO; HypAirFeNO, Fountain Valley, CA) was measured for both HDM‐SLIT and no‐AIT participants in parts per billion [ppb] and assessed following the manufacturer's instructions, immediately before treatment at t = 0 and at t = 18 months.

Blood samples were collected at t = 0, 4, 12, and 18 months in lithium‐heparinized tubes and processed within 24 h. 50 μL whole blood was used for TruCount analysis (see ***Flow cytometry*** below). Plasma was collected and stored at −80°C. Peripheral blood mononuclear cells (PBMC) were isolated with Ficoll Paque (GE Healthcare, Chicago, IL, USA) and gradient centrifugation, and subsequently stored in freezing media (50% fetal calf serum, 40% RPMI 1640, and 10% DMSO) in liquid N_2_ for later flow cytometry analysis (see below).

### 2.3 Protein Production and Tetramerization

Recombinant HDM proteins Der p 1 and Der p 2 were produced as previously described [33]. The isoforms Der p 1.0102, Der p 2.0101 were generated as recombinant proteins with the leader sequence with a 6‐His tag and an Avi‐tag in the ExpiSF9 insect cell line. Mutations were introduced in the Der p 1 construct at the site of cysteine protease activity and the N‐glycosylation motif for successful production [33]. Recombinant proteins then underwent purification and concentration. The validated proteins were subjected to targeted biotinylation with a BirA enzyme (*Escherichia coli* 6.3.4.15; Avidity LLC, Aurora, CO, US) in SuperMix buffer (10×; Avidity LLC), incubating at 4°C overnight (44), followed by dialysis against 10 mM TRIS (pH 8) for 36 h at 4°C. For flow cytometry experiments, biotinylated Der p 1 was tetramerized with either Brilliant Ultraviolet (BUV)395‐conjugated streptavidin (strep) or Brilliant Violet (BV)421‐conjugated strep, and Der p 2 was tetramerized with step‐BV480 or strep‐BUV737.

### 2.4 Quantification of Allergen‐Specific Serum IgE, IgG_2_, and IgG_4_

HDM‐specific IgE, IgG2, and IgG4 levels were measured by in‐house enzyme‐linked immunosorbent (ELISA) assays for 21 HDM‐SLIT patients and 17 no‐AIT patients at all four time points (t = 0, 4, 12, and 18 months), as described recently [21]. Briefly, plate wells were coated with HDM extract (2 μg/mL; *D. pteronyssinus* extract; Stallergenes), recombinant Der p 1 (1 μg/mL), or Der p 2 (1 μg/mL), followed by blocking with 5% skim milk powder in PBS for IgE ELISA or bovine serum albumin in PBS for IgG2 and IgG4 ELISAs. Subsequently, wells were incubated with diluted serum samples for 3 h at room temperature (RT) for IgE ELISA and for 1 h at 37°C. Standard curves were generated with IgE (clone AbD18705_hIgE; Bio‐Rad, Hercules, CA), IgG2 (clone AbD18705_hIgG2; Bio‐Rad), and IgG4 (clone AbD18705_hIgG4; Bio‐Rad). The antibodies bound to HDM extract, Der p 1, and Der p 2 were detected with polyclonal rabbit anti‐human IgE (Cat#: A009402, Dako), polyclonal mouse anti‐hIgG2 (Cat#:555,873; BD Bioscience), or biotinylated anti‐hIgG4 (Cat#: B3648, Sigma Aldrich), followed by secondary detection with goat anti‐rabbit IgG‐HRP (Cat#: W4011, Promega), goat anti‐mouse IgG1:HRP (Cat#: STAR132P, Biorad), or Pierce high sensitivity strep‐HRP (Cat#: 21130, Thermo Scientific). For Der p 1‐specific IgE measurements, the goat anti‐hIgE cross‐adsorbed secondary antibody HRP (Cat#: A18799, Invitrogen) was used for direct detection of specific IgE. ELISA was then developed with TMB (Cat#: 2023, Life Technologies, Carlsbad, CA) and stopped by HCl (1 N; Chem‐Supply). The absorbance at OD450nm from each well was measured with the Multiskan Microplate Spectrophotometer (Thermo Fisher). Wells without any allergens were used to determine the background values to be subtracted from the specific antibody IgE, IgG2, and IgG4 values, and results were expressed in optical density (OD) values for further analysis.

### 2.5 Flow Cytometry

#### 2.5.1 TruCount

Absolute numbers of B cells, T cells, NK cells, and monocytes were determined in whole blood from each sample, as described previously [34], with the TruCount Absolute Counting tubes (BD Biosciences, Franklin Lakes, NJ, USA). In short, fresh blood samples (50 μL) were stained with the antibody cocktail mix containing CD3, CD4, CD8, CD16, CD19, CD45, and CD56 (Table S2) in the dark for 15 min. Staining was stopped by adding 500 μL of BD Lysis solution (1×) to the tube, followed by analysis on the FACSLyric or LSRII (both BD Biosciences). The absolute count of each leukocyte and lymphocyte subpopulation was calculated as per the manufacturer's instructions.

#### 2.5.2 Memory B Cell Panel

Cryopreserved PBMC were thawed and washed with FACS buffer (0.7% BSA and 0.1% sodium azide in PBS), and then strained through a 70‐μm filter. Cell count and viability were determined with an automated cell counter (Nexcelom, Lawrence, MA). A total of 10–15 million PBMC were incubated with 5 μg/mL of each of the four tetramers for 3 min, followed by incubation with a cocktail of antibodies: CD3, CD19, CD21, CD23, CD27, CD29, CD38, CD45, CD124 (IL‐4Rα), IgA, IgD, IgE, IgG1, IgG2, IgG3, IgG4, IgM, and with Live dead Blue (Tables S3 and S4), in a total volume of 250 μL FACS buffer for 15 min at RT. In parallel, ~5 million PBMC were incubated with BUV395‐, BV421‐, BV480‐, and BUV737‐conjugated strep controls with the same procedure, followed by incubation with a minimal antibody backbone: CD3, CD19, and Live Dead Blue. After staining, cells were washed with FACS buffer and then fixed with 2% paraformaldehyde for 20 min at RT. Samples were then washed again and resuspended in FACS buffer for analysis on a 5‐laser Cytek Aurora (Cytek Biosciences). The standard Bmem Assay Settings were employed [35], and adjusted daily utilizing the SpectroFlo Quality Control beads. Light scatter settings were fine‐tuned for lymphocyte identification. The live unmixing function on SpectroFlo was employed, and samples were run at a flow rate of ~5,000 events per second.

Analysis of flow cytometric data was conducted on Flowjo (v10; TreeStar, Ashland, Ore). Total, Der p 1 and Der p 2‐specific Bmem were defined and immunophenotyped in detail (Figure S1). In brief, CD19^+^ B cells were gated to identify the CD38^dim^ mature B cells, and subsequently Bmem through exclusion of IgD^+^CD27^−^ naïve B cells. Allergen‐specific Bmem were defined by double discrimination with Der p 1 or Der p 2 tetramers as described previously [36]. Within the total and allergen‐specific Bmem populations, immunoglobulin isotypes and Ig subclasses were identified with the corresponding antibodies (Figure S1A–C). The CD38^dim^CD21^lo^ atypical B cell population was also defined within Bmem (Figure S1D). Absolute B cell counts per microliter of blood obtained from the TruCount tube were used to convert total and allergen‐specific B cell subset frequencies into absolute cell counts.

### 2.6 Statistical Analysis

Statistical analysis for clinical information, serology, and flow cytometric results was performed with GraphPad Prism (v9.0.1; GraphPad Software, Dotmatics, Boston, MA, USA). The nonparametric Mann–Whitney test was conducted for unpaired data, and the nonparametric Wilcoxon matched‐pairs signed‐rank test was used for paired data. The Friedman test with post hoc Wilcoxon matched‐pairs signed‐rank test was used to examine differences across timepoints from the same individual. Correlation analyses between Type 2 Bmem count and IgE serology assays were conducted using Spearman's rank correlation test to assess monotonic relationships. Subsequent post hoc analyses involved log–log non‐linear fitting, and correlation was determined by the coefficient of determination (r^2^). Power calculations were performed to ensure sample sizes of 15–21 delivered adequate power to observe clinical improvement and longitudinal immunological changes [14, 21]. For all tests, *p* < 0.05 was considered statistically significant.

## 3 Results

### 3.1 SLIT For HDM Showed Clinical Efficacy Over 18 Months

**FIGURE 1:**
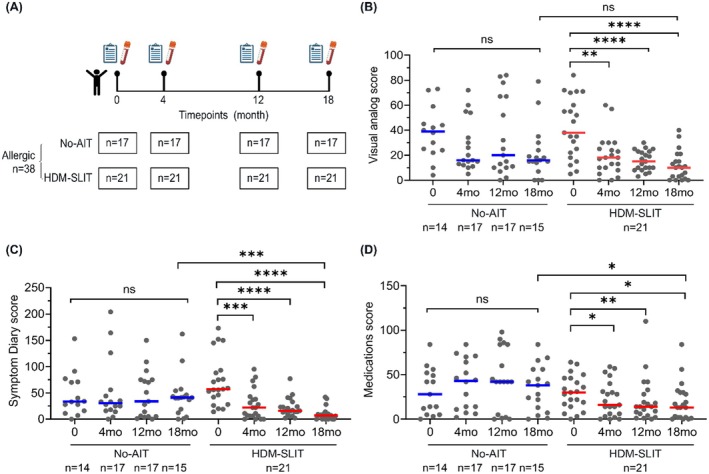
Study design and clinical parameters over the 18‐month time period. (A) Schematic outlining the patient groups and blood sample collection time points. (B) Visual analog scores, (C) Symptom diary scores, and (D) medication scores at 0, 4, 12, and 18 months for the patients without AIT (no‐AIT; blue) and patients on treatment (HDM‐SLIT; red). Individual data points are shown with median lines. Statistics: Wilcoxon signed rank test; * *p* < 0.05, **p < 0.01, ***p < 0.001, ****p < 0.0001, ns: Not significant.

A total of 38 HDM‐sensitized patients were included in the study (Table 1, Figure 1A). Of the 38 patients, 21 received 18 months of HDM‐SLIT, and 17 were treated with conventional pharmacotherapy only (No‐AIT). Sex and age distributions were similar between groups (Table 1). All 38 HDM‐allergic patients suffered from AR, and just over half of the participants in both the HDM‐SLIT (55%) and no‐AIT (56%) groups had concomitant asthma. Specific sIgE levels were consistent between the HDM‐SLIT and no‐AIT groups (Table 1).

**TABLE 1:** Demographics and comparison between no‐AIT control and HDM‐SLIT subjects.

	No‐AIT *n* = 17	HDM‐SLIT *n* = 21	No‐AIT vs. HDM‐SLIT (p value)
Female sex (*n*; %)	14 (82.3%)	12 (57.1%)	0.70
Median age (range; years)	30 (19–57)	29 (19–52)	0.88
Allergic rhinitis (*n*; %)	17 (100%)	21 (100%)	> 0.9999
Perennial	17 (100%)	21 (100%)	> 0.9999
Seasonal	13 (76.5%)	11 (52.4%)	0.1812
Asthma (*n*; %)	10 (55.6%)	12 (54.5%)	> 0.9999
Median HDM sIgE (kU_A_/L; range)	27.8 (10.1– > 100)	16.8 (5.17– > 100)	0.31
FeNO, t = 0 (ppb; median, range)	43 (9, 104)	23 (3, 66)	0.03
FeNO, t = 18 months (ppb; median, range)	21 (6, 123)^a^	24 (3, 73)^b^	0.84
FEV1, t = 0 (%; median, range)			
Pre‐bronchodilator	95 (59, 119)	96 (77, 110)^b^	0.72
Post‐bronchodilator	98 (89, 122)	98 (81, 110)^b^	0.49
FEV1, t = 18 months (%; median, range)			
Pre bronchodilator	94 (78, 120)	94 (76, 106)	0.80
Post bronchodilator	98 (87, 122)	96 (76, 105)	0.24

^a^ Data collected from 15/17 patients.

^b^ Data collected from 19/21 patients.

Sample collections for the no‐AIT group occurred slightly later at 4, 12, and 18 months than in the HDM‐SLIT group, but this did not affect the validity of immunological comparisons between groups (Table S1). Initial lung function was similar between groups, with the median forced expiratory volume one second (FEV1) being over 90% for both HDM‐SLIT and no‐AIT groups (Table 1). Airway inflammation [37], assessed by Fractional exhaled nitric oxide (FeNO), remained at a similar level at t = 0 to 18 months (median: 23 ppb‐24 ppb) for the HDM‐SLIT group, but as expected, was reduced over 18 months for the no‐AIT group (median: 43 ppb‐21 ppb), who were using standard pharmacotherapy for symptoms. Due to non‐randomized group allocation, the control group demonstrated higher baseline FeNO levels than the treatment group (Table 1). These respiratory parameters reflected that both groups of allergic patients had normal lung function with stable asthma throughout the study and received optimal medical attention and treatment over the study period (Table 1).

Visual analog score (VAS), symptom diary, and medication scores were recorded over 18 months to monitor symptoms and assess patients' life quality [38, 39]. For both groups, the median VAS reduced 4 months into treatment and remained lower than baseline at 12 and 18 months. This reduction was significant across treatment compared to t = 0 for the HDM‐SLIT group but not in the no‐AIT group (Figure 1B). Similarly, the symptom diary and medication scores were only significantly reduced over time in the HDM‐SLIT treatment, and not in the no‐AIT group. (Figure 1C,D). Overall, significant clinical improvement with reduced pharmacotherapy intake was observed at all follow‐up time points over 18 months in the HDM‐SLIT group, whereas improved VAS in the no‐AIT group was not related to reduced symptoms or medication intake.

### 3.2 AIT‐Induced Time‐Dependent Changes in Allergen‐Specific Immunoglobulins

**FIGURE 2:**
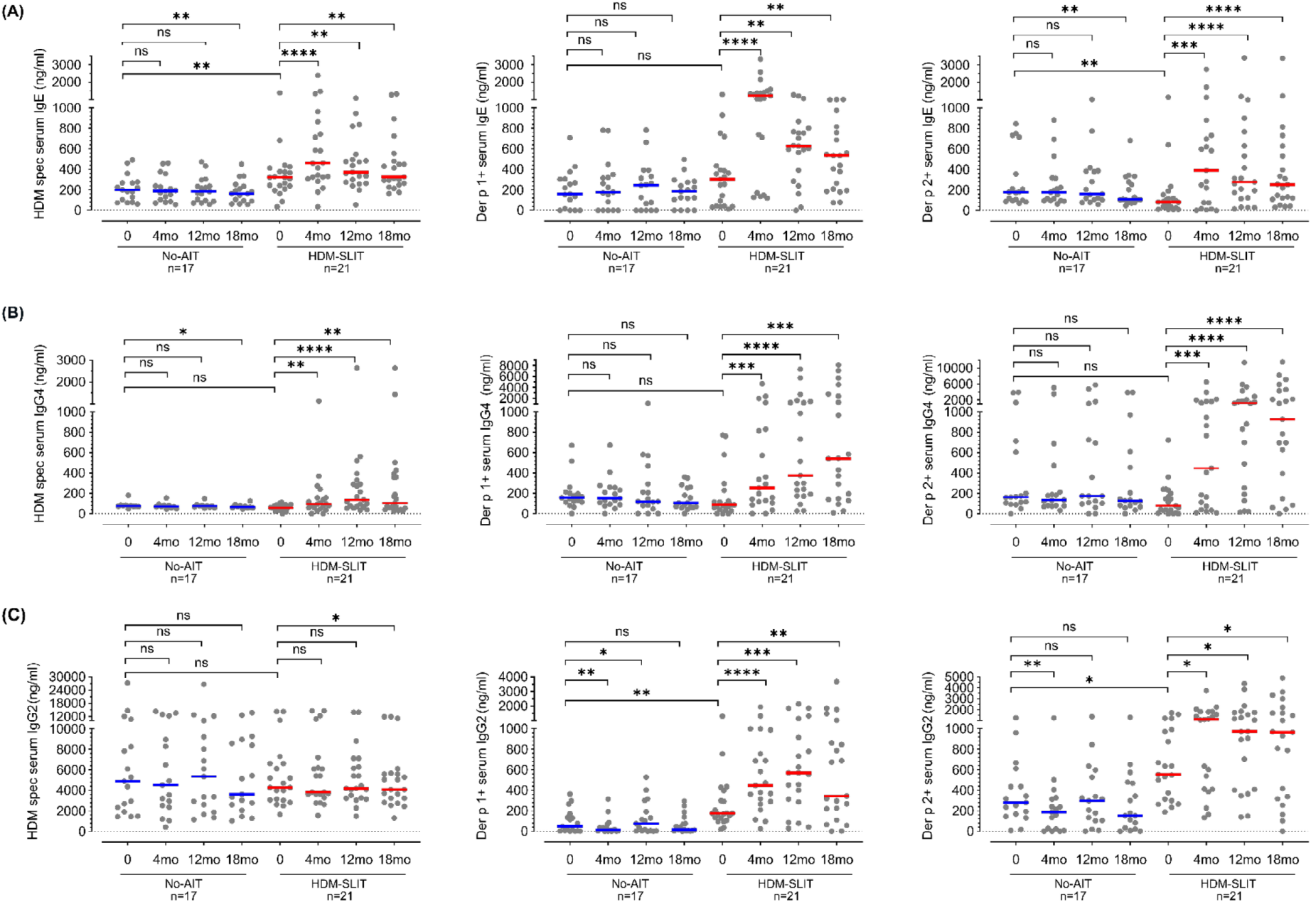
Longitudinal evaluation of serum HDM‐specific IgE, IgG4, and IgG2 levels. HDM extract‐, Der p 1‐, and Der p 2‐specific serum levels of (A) IgE, (B) IgG4, and (C) IgG2, and at t = 0, 4, 12, and 18 months for pharmacology‐only treated patients (no‐AIT; *n* = 17) and AIT‐treated patients (HDM‐SLIT; *n* = 21). Statistics: Friedman test with post hoc Dunn's multiple comparisons test for paired data across 18 months; Mann–Whitney U test for unpaired data. *p < 0.05, **p < 0.01, ***p < 0.001, ****p < 0.0001, ns: Not significant.

To evaluate the immunological effects of the observed effective AIT treatment, we examined the HDM, Der p 1, and Der p 2‐specific serum IgE, IgG4, and IgG2 levels by enzyme‐linked immunosorbent assay (ELISA) or ImmunoCAP (from the Hospital Pathology). HDM‐specific IgE levels increased following commencement of SLIT, but not in the no‐AIT group. The median IgE levels peaked at 4 months on SLIT and subsequently declined at 12 and 18 months (Figure 2A). Specific IgG4 levels substantially increased over 18 months of treatment, while no increase was seen for the no‐AIT group (Figure 2B). Serum‐specific IgG2 to HDM extract did not change significantly, whereas Der p 1 and Der p 2 specific IgG2 levels were significantly increased on AIT treatment but not in the no‐AIT group (Figure 2C). Thus, the HDM‐specific antibody response is modified in patients undergoing AIT.

### 3.3 AIT‐Induced Effects on the Immunophenotype of Bmem

**FIGURE 3:**
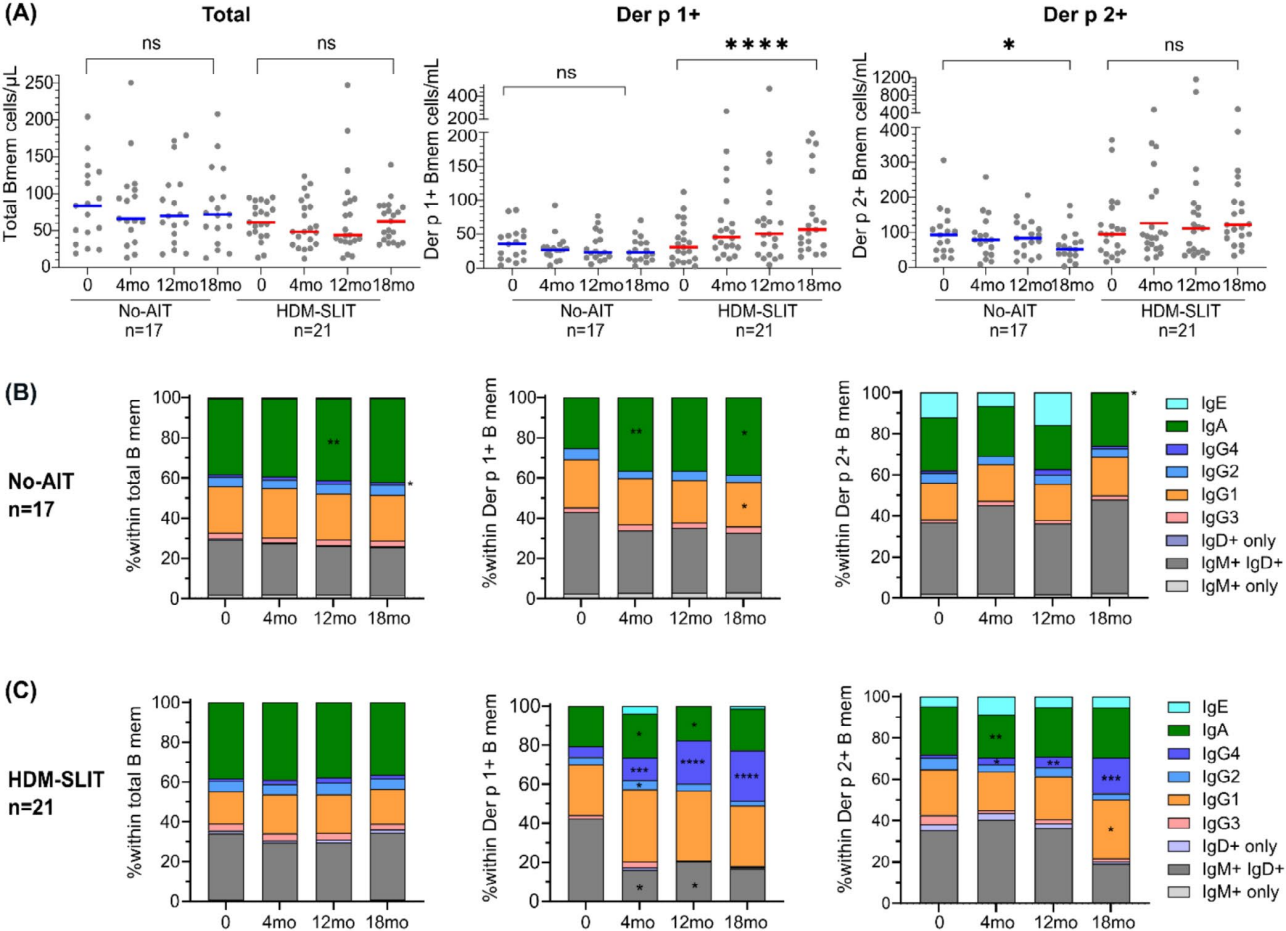
The kinetics and immunophenotypes of total, Der p 1‐ and Der p 2‐specific Bmem over 18 months with or without AIT. (A) Bmem were defined by CD27 + IgD+ and all CD27+/‐IgD‐ within CD38^dim^ cells in flow, excluding plasmablasts and naïve B cells (CD27 + IgD+). Total, Der p 1‐, and Der p 2‐specific Bmem numbers at 0, 4, 12, and 18 months in 17 no‐AIT patients (blue) and 21 HDM‐SLIT treated patients (red) are compared in parallel. Individual data points are shown with median lines. Frequencies of total, Der p 1, and Der p 2‐specific Bmem expressing IgM^+^ only, IgM^+^IgD^+^, IgD^+^ only, IgG3, IgG1, IgG2, IgG4, IgA, or IgE for (**B**) no‐AIT patients and (**C**) HDM‐SLIT treated patients at 0, 4, 12, and 18 months. Statistics: For non‐Gaussian distribution data, the non‐parametric Friedman test and/or post hoc Wilcoxon signed‐rank test was used; *p < 0.05, **p < 0.01, ***p < 0.001, ns: Not significant.

As allergen‐specific Bmem, and especially those with a type 2 phenotype, are presumed to hold immune memory for allergic responses [40], we monitored Der p 1‐ and Der p 2‐specific Bmem over 18 months of HDM‐SLIT (Figure S1A–C). Der p 1 and Der p 2 specific Bmem were defined by double discrimination using Der p 1 and Der p 2 fluorescent tetramers. In parallel, for each sample, a staining was performed with empty streptavidins. These yielded very few positive events, 8–10 fold lower than tetramer‐positive events, demonstrating the specificity of the tetramer staining (Figure S2). While absolute counts of total Bmem did not change over the course of 18 months SLIT for either treatment or control group (Figure 3A), we observed a gradual increase in Der p 1‐specific Bmem counts, which were significantly higher at 18 months than prior to SLIT treatment. Within total and allergen‐specific Bmem, subsets were defined based on distinct Ig isotype and IgG subclass expression (Figure S1A–C). Prior to SLIT, ~40% of total Bmem expressed IgM and/or IgD, with the remainder being predominantly IgG1 or IgA (~20%–40% each), and only small subsets expressing IgG2, IgG3, or IgG4. The composition of the total Bmem populations remained consistent over time in both treatment and no‐AIT groups (Figure 3B,C).

Within allergen‐specific Bmem, the populations expressing IgM and/or IgD (> 50%) and IgG1 (> 25%) were more prominent, with reduced fractions expressing IgA (< 10%) (Figure 3B,C). While the Der p 1‐specific Bmem of the no‐AIT group demonstrated a similar pattern to total Bmem, significant expansions were seen in IgG2, IgG4, and IgA expressing Der p 1‐specific Bmem since 4 months of SLIT treatment (Figure 3B,C). Notably, following the early expansion at 4 months, the IgA‐expressing Der p 1‐specific Bmem population was significantly reduced at 18 months. Within Der p 2‐specific Bmem, fractions expressing IgG1 or IgG4 were also expanded across treatment. IgA‐expressing Der p 2‐specific Bmem showed an early decrease at 4 months of treatment and subsequent return to pre‐treatment baseline. No changes were observed in atypical CD38^dim^CD21^lo^ cells within the total or allergen‐specific Bmem populations (Figure S3). Thus, HDM‐SLIT was associated with the expansion of Der p 1‐ and Der p 2‐specific Bmem with increased proportions expressing either IgG4 or IgA.

### 3.4 AIT Modified Type 2 Bmem With Expression of Potential Surface Biomarkers

**FIGURE 4:**
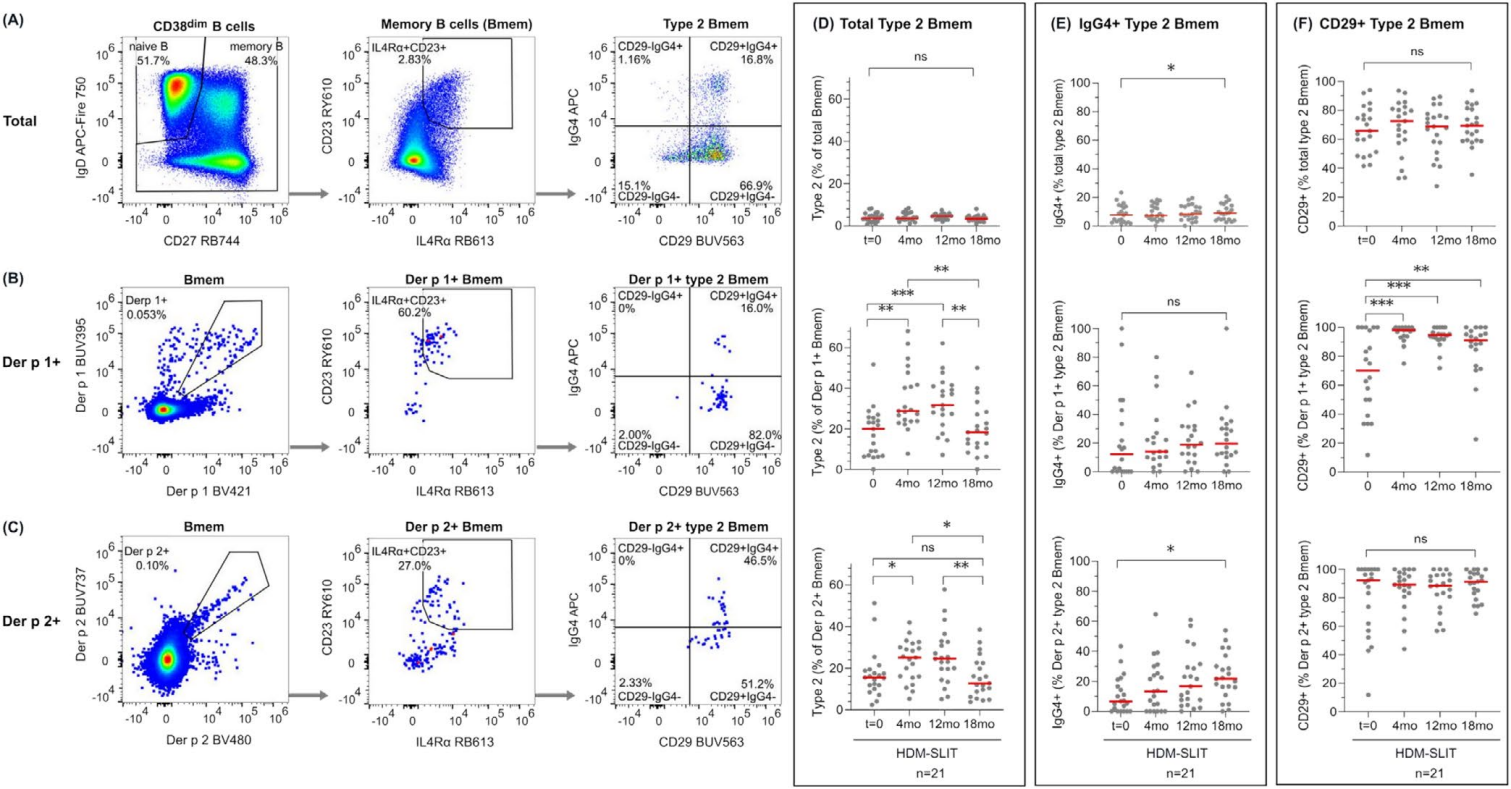
Numbers and immunophenotype of allergen‐specific type 2 Bmem during HDM‐SLIT. Bmem were defined within CD38^dim^ B cells through the exclusion of naive B cells (CD27^−^IgD^+^) (Figure S1). Within (A) total, (B) Der p 1‐ and (C) Der p 2‐specific Bmem, IL4Rα^+^ CD23^+^ type 2 Bmem were defined. Within type 2 Bmem, cells expressing CD29 and/or IgG4 were defined. (D) Frequencies of type 2 Bmem within the total, Der p 1, and Der p 2‐specific Bmem populations over the 18‐month HDM‐SLIT. (E) Frequencies of cells expressing IgG4, or (F) expressing CD29 within the total, Der p 1 and Der p 2‐specific type 2 Bmem before and on HDM‐SLIT. For the Der p 1‐specific type 2 Bmem data on panels B–D, one sample was excluded with too few Type 2 Bmem events at t = 0 and t = 18 months measurements. Single data points are shown with red lines in panels D–F. Statistics: The non‐parametric Friedman test and/or post hoc Wilcoxon matched‐pairs signed‐rank test, w; *p < 0.05; **p < 0.01; ***p < 0.001; ns, not significant.

Within the allergen‐specific Bmem, the Type 2 subset was defined based on co‐expression of CD23 and CD124 (IL‐4Rα) (Figure 4A–C) [23, 24]. In allergic patients pre‐AIT, a significantly higher proportion of Der p 1‐ and Der p 2‐specific Bmem displayed a type 2 phenotype than their total Bmem population (Figure 4D). Over time in the absence of AIT, total and allergen‐specific type 2 Bmem frequencies did not change (Suppl. Figure 4), and neither did total type 2 Bmem frequencies during AIT (Figure 4D). In contrast, within both Der p 1 and Der p 2 specific Bmem, the Type 2 subset proportionally increased at 4 and 12 months on AIT, followed by a marked decline at 18 months (Figure 4D).

As Der p 1‐ and Der p 2‐specific Type 2 Bmem proportions changed during AIT, we examined relevant phenotypic dynamics in this subset (Figure S5) [41]. Within total Type 2 Bmem of patients on SLIT, frequencies expressing IgG4 significantly increased over 18 months (Figure 4E). While no changes were observed for IgG4 on Der p 1‐specific Type 2 Bmem, the proportions of IgG4^+^ Der p 2‐specific Type 2 Bmem gradually increased and were significantly higher at 18 months (*p* < 0.05). In addition, significantly higher proportions of Der p 1‐specific Type 2 Bmem expressed CD29 as early as 4 months after SLIT initiation to the end of the study at 18 months (Figure 4F).

To further explore whether increased expression of IgG4 and CD29 on Type 2 Bmem was associated with clinical improvement, correlations were made with the ratios of clinical scores at 18 vs. 0 months. No significant correlations were found for CD29 expression and clinical scores. In contrast, a significant inverse correlation was found for the ratios of IgG4 expression on both Der p 1‐ and on Der p 2‐specific Type 2 Bmem and symptom scores (Spearman's rho = −0.5794, *p* = 0.0413; Spearman's rho = −0.67881, *p* = 0.0073) (Figure S6). Overall, during AIT, there is an early and marked expansion of allergen‐specific Type 2 Bmem, but these cells are phenotypically modified with increased expression of IgG4 and CD29; this initial expansion and change is followed by a reduction in abundance at 18 months.

## 4 Discussion

We performed a longitudinal evaluation of allergen‐specific Type 2 Bmem over 18 months in HDM allergic patients either undergoing HDM‐SLIT or treated with standard pharmacotherapy. During AIT, a biphasic response was observed in allergen‐specific Type 2 Bmem with population expansion and early phenotypic changes at 4 and 12 months, followed by depletion of the Type 2 Bmem population by 18 months.

The patients in the HDM‐SLIT and no‐AIT groups were recruited from a single teaching hospital on the basis of having AR and HDM sensitization, with or without atopic asthma. With about 55% of AR patients suffering from asthma and with more females than males, the population reflects those from previous studies [42, 43]. The higher baseline FeNO in the no‐AIT group may indicate higher levels of upper or lower airway inflammation, which could potentially influence their clinical outcomes over the course of the study but would not influence the longitudinal phenotypic changes observed in the HDM‐SLIT group. The treatment group benefited over the conventional therapy group from HDM‐SLIT with an early and maintained reduction in symptoms and reduced need for additional medication; this was associated with increased serum specific IgG4 and IgG2 levels. Overall, the observed clinical benefit and immunological changes indicated successful treatment [12, 20, 44, 45], making this cohort a relevant study population for in‐depth immunological studies into the reshaping of immune memory following AIT.

Recombinant Der p 1 and Der p 2 allergens were used to evaluate allergen‐specific immune responses driven by SLIT [46]. Der p 1 and Der p 2 are major HDM allergens, with 95% of HDM allergic patients showing IgE reactivity against either or both components [8, 47]. All patients in our cohort were sensitized to either or both components, and the serum‐specific IgE to both components increased early during treatment. Similarly, IgG2 and IgG4 to both Der p 1 and Der p 2 increased, whereas this was minimal for HDM extract, which contains many non‐allergic components [46]. We previously demonstrated that fluorescent tetramers of Der p 1 and Der p 2 allowed sensitive and specific determination of allergen sensitization by flow cytometry on basophils [33]. Importantly, inclusion of both allergen components also provided the opportunity to evaluate in parallel Bmem populations with distinct specificities, yet derived from the same allergen. This enabled a comprehensive understanding of the effects of AIT on Bmem in relation to disease remission.

On treatment, Der p 1‐ and Der p 2‐specific Bmem numbers expanded, and greater proportions utilized IgG2 and IgG4, encoded by more downstream‐located constant regions in the Ig heavy chain locus [48]. This expands our previous observations showing similar patterns early on during AIT for grass pollen and bee venom [21, 22]. Following bee venom subcutaneous AIT, Api m 1‐specific B cell receptors were directed to more diverse epitopes [21], indicating clonotype shifts in the composing population were likely. It may be that HDM‐SLIT also induces such epitope spreading, which may contribute to more effective blocking of IgE binding to HDM allergens, or alternatively, the SLIT‐AIT may have reprogrammed the same clones to different functionality. Either mechanism could underlie the clinical benefits observed in the early stages (4 and 12 months) of SLIT, and as the reservoir of allergen‐reactive antibody‐producing cell precursors, may be important in driving sustained non‐responsiveness at 18 months and beyond.

In addition to increased downstream IgG subclass usage, more allergen‐specific Bmem expressed IgA during HDM‐SLIT. Previous studies have reported increased serum‐specific IgA during SLIT for grass pollen and HDM allergies [49, 50, 51, 52, 53]. Similar to IgG2 and IgG4, the IgA‐encoding constant regions are located downstream of IgG1 [48]. Our data suggest that 18 months SLIT drives class‐switching downstream Ig constant regions encoding antibodies with more regulatory capacities.

In line with previous observations for other allergies [23, 24, 25], we found that in HDM allergic patients prior to AIT, a significantly higher proportion of allergen‐specific Bmem has a type 2 phenotype compared to the total Bmem population. Type 2 Bmem express germline *IGHE* transcripts [22, 23, 24], and can readily differentiate into IgE‐producing cells, acting as the reservoir of pathogenic IgE [26]. Our findings expand on previous findings reports of increased allergen‐specific Type 2 Bmem in patients with ryegrass pollen allergy, bee venom allergy, and peanut allergy [23, 24, 25], strengthening the reported association between type 2 Bmem and allergen sensitization [23, 24, 54].

Consistent with our previous RGP and bee venom AIT findings [21, 25], we here in HDM‐AIT also observed increased frequencies of Der p 1‐specific type 2 Bmem expressing CD29 after several months of SLIT. In addition, we found that this increase was consistent over 18 months of treatment. Interestingly, while most individuals showed reductions at 18 months, two individuals persisted with high Type 2 Bmem representation; their clinical symptoms were still diminished relative to pre‐AIT, but it is enticing to consider whether the durability of the non‐responsiveness would differ in these individuals off‐therapy relative to those with a diminished mBC2 representation at this time. CD29 negatively regulates BCR signaling, and increased expression of CD29 may inhibit the B‐cell response upon re‐exposure [27, 55], potentially contributing to immunological tolerance to the presented allergen. It remains unknown whether the increased expression of IgG4 and CD29 is dependent on ongoing AIT or is simply a marker of a composition shift. We favor the view that this change in Type 2 Bmem phenotype is plastic and depends on the continued allergen exposure, so it operates as a marker of recent re‐exposure rather than a conserved change in Bmem state.

To determine the clinical predictive value of the Type 2 Bmem biomarkers, future research is required to reliably detect these rare cell populations. Key considerations include overcoming technical constraints in flow panels and the acquisition of high cell numbers. Ultimately, validation in larger, real‐world studies is needed to identify if the Type 2 Bmem dynamics can discriminate between responders and non‐responders.

The AIT‐driven decrease of allergen‐specific Type 2 Bmem by 18 months may suggest the reshaping of immune memory, and explain the observed reduced production of allergen‐specific IgE at that time. As total allergen‐specific Bmem do not decline, it is possible that type 2 Bmem change their phenotype into conventional Bmem, or that these are depleted and new, conventional Bmem are formed during AIT. The serum specific IgE levels mirrored the biphasic Type 2 Bmem numbers, and following a peak at 4 months, these titers declined at 12 and 18 months of AIT. Sustained clinical improvements were also observed following 18 months in randomized clinical trials of HDM SLIT [5, 6]. In real‐world situations, long‐term course (≥ 18 months) and treatment compliance may be essential to induce immune memory changes required for sustained clinical benefit [56]. As the patients in our cohort reported clinical improvement over the full 18 months of treatment, we conclude that the immune modulation of AIT has 2 phases: (1) Expansion of allergen‐specific Type 2 Bmem with the increased expression of CD29 and IgG4 that occurs as a mechanism independent to the alleviation of symptoms; (2) Decline of Type 2 Bmem, accompanied by decreased IgE production. Therefore, our study prompts follow up study to at least 36 months to investigate these immunological changes and the long‐term Type 2 Bmem modulation in potential clinical remission.

Licensed AIT therapies include treatment durations of 36 months or longer, so a natural outstanding question in this space is for evaluation of Bmem composition and clinical state off therapy. If indeed allergen‐specific Type 2 Bmem need to be depleted for sustained unresponsiveness, this process could be expedited when AIT is combined with additional therapeutics. Options could include dupilumab (anti‐IL‐4Rα antibody), which was shown to reduce type 2 Bmem frequencies and pathogenic IgE production over time [57]; or anti‐IL21 antibody, which, through its general impacts on Bmem formation, can reduce formation of mature type 2 Bmem [58]. Indeed, a recent publication shows that allergen re‐challenge in mice in the context of anti‐IL‐4Rα treatment, or with a Th1‐promoting adjuvant, can shift the type 2 Bmem compartment towards a ‘tolerant’ IgG2c‐dominated cell pool [59]. Interventions that expedite conversion of type 2 Bmem to tolerizing Bmem are indicated as a strong means to resolve protracted allergies.

## Author Contributions

Conceptualization: MCvZ, REOH, M.H., L.M. Methodology: MCvZ, REOH, M.H., P.M.H., L.H. Investigation: L.H., S.R., AvB, P.M.A., K.D. Funding acquisition: MCvZ, REOH, M.H., L.M. Supervision: MCvZ, REOH, AvB. Writing – Original draft: L.H., MCvZ. Writing – Review and editing: S.R., AvB, P.M.A., K.D., P.M.H., L.M., M.H., REOH.

## Funding

This study was supported by an NHMRC Ideas Grant (#2000773) to MCvZ, REO'H, and M.H., and an investigator‐initiated grant from Stallergenes‐Greer.

## Conflicts of Interest

MCvZ and REO'H are inventors on a patent related to this work (PCT/AU2023/050439). All other authors declare no conflicts of interest.

## Supporting information


**Figure S1:** Allergen‐specific Bmem gating strategy. (A) From all events, the singlet live cells were gated, with subsequent gating of the lymphocyte population using CD45 and SSC. CD3^+^ T and CD19^+^ B‐cells were discriminated within lymphocytes, followed by the subsetting of B cells into CD38^dim^ mature B cells, transitional B cells, and plasmablasts. Within the CD38^dim^ cells, Bmem were defined by excluding naive B cells (CD27‐IgD+). Bmem were separated using IgM and IgD into unswitched Bmem (IgM+ only, IgD+ only, and IgM + IgD+) and Immunoglobulin (Ig) class‐switched populations. Ig‐switched subsets were then subsetted based on the differential expression of IgG1, IgG2, IgG3, IgG4, IgA, and IgE. (B) The detection of Der p 1 and (C) Der p 2 specific cells within Bmem and subsequent evaluation of Ig isotype and IgG subclass expression, as in panel A. (D) Within the total, Der p 1 and Der p 2 specific Bmem, the CD38^dim^CD21^lo^ atypical Bmem population was defined.
**Figure S2:** Der p 1 and Der p 2 tetramer staining and streptavidin‐only control double‐discrimination‐stained Bmem in the treatment group Representative plots from three participants on (A) Der p 1‐ and (B) Der p 2‐ tetramer staining compared to streptavidin‐only control at t = 0 and t = 18 of SLIT treatment.
**Figure S3:** CD38^dim^CD21^lo^ expressing‐ allergen‐specific Bmem over 18‐months in patients without or with SLIT. Frequencies of CD38^dim^CD21^lo^ events within the (A) total, (B) Der p 1, and (C) Der p 2 Bmem. Individual data points are shown with median lines: Blue, no‐AIT; red, HDM‐SLIT. Statistics: The non‐parametric Friedman test and/or post hoc Dunn's multiple comparisons test; *p < 0.05.
**Figure S4:** Numbers and immunophenotypes of allergen‐specific type 2 Bmem of the no‐AIT patient group. Bmem were defined within CD38^dim^ B cells through the exclusion of naive B cells (CD27^−^ IgD^+^; Fig. S1). Within (A) total, Der p 1‐ and Der p 2‐specific Bmem, IL4R^+^CD23^+^ type 2 Bmem were defined. Within type 2 Bmem, cells expressing (B) IgG4 and (C) CD29 were defined. Single data points are shown with blue lines denoting median values. Statistics: Normality Shapiro–Wilk tests. Data followed a non‐Gaussian distribution; the non‐parametric Friedman test and/or post hoc Wilcoxon matched‐pairs signed‐rank test was used; *p < 0.05; ns, not significant.
**Figure S5:** Kinetics of the frequency of the total and allergen‐specific Type 2 Bmem and the subsets expressing IgG4+ and CD29+ across 18 months of treatment. (A) The kinetics of the frequencies of the total, Der p 1 and Der p 2 (A) Type 2 Bmem (B) IgG4‐expressing Type 2 Bmem, and (C) CD29‐expressing Type 2 Bmem across 18 months of treatment in HDM‐SLIT subjects. Statistics: The non‐parametric Friedman test and/or post hoc Wilcoxon matched‐pairs signed‐rank test, w; *p < 0.05; **p < 0.01; ***p < 0.001; ns, not significant.
**Figure S6:** Correlation analysis of the change in clinical symptoms with the change in allergen‐specific IgG4+ Type 2 Bmem. Ratios of the frequencies of IgG4^+^ events 18 months over 0 months within (A) Der p 1‐ and (B) Der p 2‐specific Type 2 Bmem (IL4Rα^+^ CD23^+^) plotted vs. the ratio of the symptom scores at 18 over 0 months of treatment. Individual data points are shown with linear regression lines. Sample sizes for each measurement are indicated, where samples with zero events of specific Type 2 Bmem were excluded from analysis. Spearman's rank correlation tests were conducted, with the rho correlation coefficient indicating the monotonic relationship between the subset and clinical parameter, and the *p*‐values indicating the significance of the correlation tests.
**Table S1:** Timing of sample collections.
**Table S2:** List of antibodies used for Trucount analysis.
**Table S3:** List of antibodies used in memory B cell flow cytometry.
**Table S4:** Composition of the antibody panels.

## Data Availability

The data that support the findings of this study are available from the corresponding author upon reasonable request.

## References

[1] J. B. Soriano , P. J. Kendrick , K. R. Paulson , et al., “Prevalence and Attributable Health Burden of Chronic Respiratory Diseases, 1990–2017: A Systematic Analysis for the Global Burden of Disease Study 2017,” Lancet Respiratory Medicine 8, no. 6 (2020): 585–596. 32526187 10.1016/S2213-2600(20)30105-3 PMC7284317

[2] S. R. Durham and M. H. Shamji , “Allergen Immunotherapy: Past, Present and Future,” Nature Reviews Immunology 23, no. 5 (2023): 317–328. 10.1038/s41577-022-00786-1 PMC9575636 36253555

[3] J. C. Virchow , V. Backer , P. Kuna , et al., “Efficacy of a House Dust Mite Sublingual Allergen Immunotherapy Tablet in Adults With Allergic Asthma: A Randomized Clinical Trial,” JAMA 315, no. 16 (2016): 1715–1725. 27115376 10.1001/jama.2016.3964

[4] B. Fritzsching , M. Contoli , C. Porsbjerg , et al., “Long‐Term Real‐World Effectiveness of Allergy Immunotherapy in Patients With Allergic Rhinitis and Asthma: Results From the REACT Study, a Retrospective Cohort Study,” Lancet Regional Health–Europe 13 (2022): 100275. 34901915 10.1016/j.lanepe.2021.100275 PMC8640513

[5] M. Penagos and S. R. Durham , “Allergen Immunotherapy for Long‐Term Tolerance and Prevention,” Journal of Allergy and Clinical Immunology 149, no. 3 (2022): 802–811. 35085663 10.1016/j.jaci.2022.01.007

[6] K.‐C. Bergmann , P. Demoly , M. Worm , et al., “Efficacy and Safety of Sublingual Tablets of House Dust Mite Allergen Extracts in Adults With Allergic Rhinitis,” Journal of Allergy and Clinical Immunology 133, no. 6 (2014): 1608–1614.e1606. 24388010 10.1016/j.jaci.2013.11.012

[7] M. A. Calderón , A. Linneberg , J. Kleine‐Tebbe , et al., “Respiratory Allergy Caused by House Dust Mites: What Do We Really Know?,” Journal of Allergy and Clinical Immunology 136, no. 1 (2015): 38–48. 25457152 10.1016/j.jaci.2014.10.012

[8] G. Pittner , S. Vrtala , W. R. Thomas , et al., “Component‐Resolved Diagnosis of House‐Dust Mite Allergy With Purified Natural and Recombinant Mite Allergens,” Clinical and Experimental Allergy 34, no. 4 (2004): 597–603. 15080813 10.1111/j.1365-2222.2004.1930.x

[9] T. Batard , W. G. Canonica , O. Pfaar , et al., “Current Advances in House Dust Mite Allergen Immunotherapy (AIT): Routes of Administration, Biomarkers and Molecular Allergen Profiling,” Molecular Immunology 155 (2023): 124–134. 36806944 10.1016/j.molimm.2023.02.004

[10] T. Boonpiyathad , W. van de Veen , O. Wirz , et al., “Role of Der p 1–Specific B Cells in Immune Tolerance During 2 Years of House Dust Mite–Specific Immunotherapy,” Journal of Allergy and Clinical Immunology 143, no. 3 (2019): 1077–1086.e1010. 30529452 10.1016/j.jaci.2018.10.061

[11] M. Hoshino , K. Akitsu , J. Ohtawa , and K. Kubota , “Long‐Term Efficacy of House Dust Mite Sublingual Immunotherapy on Clinical and Pulmonary Function in Patients With Asthma and Allergic Rhinitis,” Journal of Allergy and Clinical Immunology: Global 3, no. 2 (2024): 100206. 38328802 10.1016/j.jacig.2024.100206 PMC10847160

[12] A. Tanaka , Y. Tohda , K. Okamiya , R. Azuma , I. Terada , and M. Adachi , “Efficacy and Safety of HDM SLIT Tablet in Japanese Adults With Allergic Asthma,” Journal of Allergy and Clinical Immunology 8, no. 2 (2020): 710–720.e714. 10.1016/j.jaip.2019.09.002 31541768

[13] R. Varona , T. Ramos , M. M. Escribese , et al., “Persistent Regulatory T‐Cell Response 2 Years After 3 Years of Grass Tablet SLIT: Links to Reduced Eosinophil Counts, sIgE Levels, and Clinical Benefit,” Allergy 74, no. 2 (2019): 349–360. 30003552 10.1111/all.13553 PMC6585999

[14] J. J. Heeringa , C. I. McKenzie , N. Varese , et al., “Induction of IgG2 and IgG4 B‐Cell Memory Following Sublingual Immunotherapy for Ryegrass Pollen Allergy,” Allergy 75, no. 5 (2020): 1121–1132. 31587307 10.1111/all.14073 PMC7317934

[15] M. Shamji , J. Kappen , M. Akdis , et al., “Biomarkers for Monitoring Clinical Efficacy of Allergen Immunotherapy for Allergic Rhinoconjunctivitis and Allergic Asthma: An EAACI Position Paper,” Allergy 72, no. 8 (2017): 1156–1173. 28152201 10.1111/all.13138

[16] M. Shamji , C. Ljørring , J. Francis , et al., “Functional Rather Than Immunoreactive Levels of IgG4 Correlate Closely With Clinical Response to Grass Pollen Immunotherapy,” Allergy 67, no. 2 (2012): 217–226. 22077562 10.1111/j.1398-9995.2011.02745.x

[17] M. Feng , X. Zeng , Q. Su , et al., “Allergen Immunotherapy–Induced Immunoglobulin G4 Reduces Basophil Activation in House Dust Mite–Allergic Asthma Patients,” Frontiers in Cell and Developmental Biology 8 (2020): 30. 32154245 10.3389/fcell.2020.00030 PMC7044416

[18] R. Aalberse , S. Stapel , J. Schuurman , and T. Rispens , “Immunoglobulin G4: an odd antibody,” Clinical & Experimental Allergy 39, no. 4 (2009): 469–477. 19222496 10.1111/j.1365-2222.2009.03207.x

[19] D. MacGlashan, Jr. , S. Alvarez‐Arango , and J. Tversky , “Subclasses of Allergen‐Specific IgG: Serum IgG2 and IgG3 Levels Are Not Predicted by IgG1/IgG4 Levels,” Clinical and Experimental Allergy: Journal of the British Society for Allergy and Clinical Immunology 51, no. 8 (2021): 1093–1095. 34192382 10.1111/cea.13977 PMC9235034

[20] V. Bordas‐Le Floch , N. Berjont , T. Batard , et al., “Coordinated IgG2 and IgE Responses as a Marker of Allergen Immunotherapy Efficacy,” Allergy 77 (2021): 1263–1273. 34551124 10.1111/all.15107

[21] C. I. McKenzie , S. Reinwald , B. Averso , et al., “Subcutaneous Immunotherapy for Bee Venom Allergy Induces Epitope Spreading and Immunophenotypic Changes in Allergen‐Specific Memory B Cells,” Journal of Allergy and Clinical Immunology 154, no. 6 (2024): 1511–1522. 39218358 10.1016/j.jaci.2024.08.019

[22] C. I. McKenzie , N. Varese , P. M. Aui , et al., “RNA Sequencing of Single Allergen‐Specific Memory B Cells After Grass Pollen Immunotherapy: Two Unique Cell Fates and CD29 as a Biomarker for Treatment Effect,” Allergy 78, no. 3 (2023): 822–835. 36153670 10.1111/all.15529 PMC10952829

[23] J. F. Koenig , N. P. H. Knudsen , A. Phelps , et al., “Type 2–Polarized Memory B Cells Hold Allergen‐Specific IgE Memory,” Science Translational Medicine 16, no. 733 (2024): eadi0944. 38324637 10.1126/scitranslmed.adi0944

[24] M. Ota , K. B. Hoehn , W. Fernandes‐Braga , et al., “CD23+ IgG1+ Memory B Cells Are Poised to Switch to Pathogenic IgE Production in Food Allergy,” Science Translational Medicine 16, no. 733 (2024): eadi0673. 38324641 10.1126/scitranslmed.adi0673 PMC11008013

[25] A. von Borstel , S. Reinwald , P. M. Aui , et al., “Expansion of Phenotypically Modified Type 2 Memory B Cells After Allergen Immunotherapy,” Allergy 80, no. 3 (2025): 867–869. 39268605 10.1111/all.16320 PMC11891399

[26] A. von Borstel , R. E. O'Hehir , and M. C. van Zelm , “IgE in Allergy: It Takes Two,” Science Translational Medicine 16, no. 733 (2024): eadl1202. 38324640 10.1126/scitranslmed.adl1202

[27] V. Andreani , S. Ramamoorthy , R. Fässler , and R. Grosschedl , “Integrin β1 regulates marginal zone B cell differentiation and PI3K signaling,” Journal of Experimental Medicine 220, no. 1 (2022): e20220342. 36350325 10.1084/jem.20220342 PMC9814157

[28] J. A. Layhadi , A. Lalioti , E. Palmer , M. C. van Zelm , E. Wambre , and M. H. Shamji , “Mechanisms and Predictive Biomarkers of Allergen Immunotherapy in the Clinic,” Journal of Allergy and Clinical Immunology: In Practice 12, no. 1 (2024): 59–66. 37996041 10.1016/j.jaip.2023.11.027

[29] M. C. van Zelm , R. E. O'Hehir , and C. I. McKenzie , “A Recent Patent in Allergy & Immunology: Biomarkers on Allergen‐Specific Memory B Cells to Predict Allergen Immunotherapy Outcome,” Allergy 79, no. 8 (2024): 2295–2297. 38979794 10.1111/all.16238

[30] L. Hsin , M. Hew , P. M. Aui , et al., “A Single Multiplex CytoBas Assay Incorporating Eight Major Components for Accurate Detection of Allergen Sensitization in Asthma and Allergic Rhinitis,” Allergy 80, no. 4 (2025): 1047–1059. 40052465 10.1111/all.16513 PMC11969309

[31] J. Matejka , M. Glueck , T. Grossman , and G. Fitzmaurice , “The Effect of Visual Appearance on the Performance of Continuous Sliders and Visual Analogue Scales,” Paper Presented at: Proceedings of the 2016 CHI Conference on Human Factors in Computing Systems (2016): 5421–5432.

[32] R. E. O'Hehir , N. P. Varese , K. Deckert , et al., “Epidemic Thunderstorm Asthma Protection With Five‐Grass Pollen Tablet Sublingual Immunotherapy: A Clinical Trial,” American Journal of Respiratory and Critical Care Medicine 198, no. 1 (2018): 126–128. 29461859 10.1164/rccm.201711-2337LE

[33] L. Hsin , N. Varese , P. M. Aui , et al., “Accurate Determination of House Dust Mite Sensitization in Asthma and Allergic Rhinitis Through Cytometric Detection of Der p 1 and Der p 2 Binding on Basophils (CytoBas),” Journal of Allergy and Clinical Immunology 153, no. 5 (2024): 1282–1291. 38360181 10.1016/j.jaci.2024.02.002

[34] G. E. Hartley , E. S. J. Edwards , P. M. Aui , et al., “Rapid Generation of Durable B Cell Memory to SARS‐CoV‐2 Spike and Nucleocapsid Proteins in COVID‐19 and Convalescence,” Science Immunology 5, no. 54 (2020): eabf8891. 33443036 10.1126/sciimmunol.abf8891 PMC7877496

[35] J. M. Edwards , M. C. Andrews , H. Burridge , et al., “Design, Optimisation and Standardisation of a High‐Dimensional Spectral Flow Cytometry Workflow Assessing T‐Cell Immunophenotype in Patients With Melanoma,” Clinical & Translational Immunology 12, no. 9 (2023): e1466. 37692904 10.1002/cti2.1466 PMC10484688

[36] M. C. van Zelm , C. I. McKenzie , N. Varese , J. M. Rolland , and R. E. O'Hehir , “Recent Developments and Highlights in Immune Monitoring of Allergen Immunotherapy,” Allergy 74, no. 12 (2019): 2342–2354. 31587309 10.1111/all.14078

[37] L. Bjermer , K. Alving , Z. Diamant , et al., “Current Evidence and Future Research Needs for FeNO Measurement in Respiratory Diseases,” Respiratory Medicine 108, no. 6 (2014): 830–841. 24636813 10.1016/j.rmed.2014.02.005

[38] N. Crichton , “Visual analogue scale (VAS),” Journal of Clinical Nursing 10, no. 5 (2001): 706.

[39] L. Klimek , K.‐C. Bergmann , T. Biedermann , et al., “Visual Analogue Scales (VAS): Measuring Instruments for the Documentation of Symptoms and Therapy Monitoring in Cases of Allergic Rhinitis in Everyday Health Care: Position Paper of the German Society of Allergology (AeDA) and the German Society of Allergy and Clinical Immunology (DGAKI), ENT Section, in Collaboration With the Working Group on Clinical Immunology, Allergology and Environmental Medicine of the German Society of Otorhinolaryngology, Head and Neck Surgery (DGHNOKHC),” Allergo Journal International 26 (2017): 16–24. 28217433 10.1007/s40629-016-0006-7 PMC5288410

[40] J.‐S. He , S. Subramaniam , V. Narang , et al., “IgG1 Memory B Cells Keep the Memory of IgE Responses,” Nature Communications 8, no. 1 (2017): 641. 10.1038/s41467-017-00723-0 PMC5608722 28935935

[41] J. Kappen , Z. Diamant , I. Agache , et al., “Standardization of Clinical Outcomes Used in Allergen Immunotherapy in Allergic Asthma: An EAACI Position Paper,” Allergy 78, no. 11 (2023): 2835–2850. 37449468 10.1111/all.15817

[42] Australia A. National Asthma Council Australia , “The hidden cost of asthma. 2015,”.

[43] A. Acevedo‐Prado , T. Seoane‐Pillado , A. López‐Silvarrey‐Varela , et al., “Association of Rhinitis With Asthma Prevalence and Severity,” Scientific Reports 12, no. 1 (2022): 6389. 35430600 10.1038/s41598-022-10448-w PMC9013347

[44] E. Potapova , V. Bordas‐Le Floch , T. Schlederer , et al., “Molecular Reactivity Profiling Upon Immunotherapy With a 300 IR Sublingual House Dust Mite Tablet Reveals Marked Humoral Changes Towards Major Allergens,” Allergy 77, no. 10 (2022): 3084–3095. 35474582 10.1111/all.15327

[45] A. G. de Boer , J. J. van Lanschot , P. F. Stalmeier , et al., “Is a Single‐Item Visual Analogue Scale as Valid, Reliable and Responsive as Multi‐Item Scales in Measuring Quality of Life?,” Quality of Life Research 13 (2004): 311–320. 15085903 10.1023/B:QURE.0000018499.64574.1f

[46] M. C. van Zelm , M. K. CI , N. Varese , J. M. Rolland , and R. E. O'Hehir , “Advances in Allergen‐Specific Immune Cell Measurements for Improved Detection of Allergic Sensitization and Immunotherapy Responses,” Allergy 76, no. 11 (2021): 3374–3382. 34355403 10.1111/all.15036

[47] H.‐J. Huang , E. Sarzsinszky , and S. Vrtala , “House Dust Mite Allergy: The Importance of House Dust Mite Allergens for Diagnosis and Immunotherapy,” Molecular Immunology 158 (2023): 54–67. 37119758 10.1016/j.molimm.2023.04.008

[48] B. G. De Jong , H. IJspeert , L. Marques , et al., “Human IgG2‐and IgG4‐Expressing Memory B Cells Display Enhanced Molecular and Phenotypic Signs of Maturity and Accumulate With Age,” Immunology and Cell Biology 95, no. 9 (2017): 744–752. 28546550 10.1038/icb.2017.43 PMC5636940

[49] M. Sugimoto , N. Kamemura , M. Nagao , et al., “Differential Response in Allergen‐Specific IgE, IgGs, and IgA Levels for Predicting Outcome of Oral Immunotherapy,” Pediatric Allergy and Immunology 27, no. 3 (2016): 276–282. 26764899 10.1111/pai.12535

[50] J. N. Francis , L. K. James , G. Paraskevopoulos , et al., “Grass Pollen Immunotherapy: IL‐10 Induction and Suppression of Late Responses Precedes IgG4 Inhibitory Antibody Activity,” Journal of Allergy and Clinical Immunology 121, no. 5 (2008): 1120–1125.e1122. 18374405 10.1016/j.jaci.2008.01.072

[51] M. H. Shamji , J. Kappen , H. Abubakar‐Waziri , et al., “Nasal Allergen‐Neutralizing IgG4 Antibodies Block IgE‐Mediated Responses: Novel Biomarker of Subcutaneous Grass Pollen Immunotherapy,” Journal of Allergy and Clinical Immunology 143, no. 3 (2019): 1067–1076. 30445057 10.1016/j.jaci.2018.09.039

[52] M. H. Shamji , D. Larson , A. Eifan , et al., “Differential Induction of Allergen‐Specific IgA Responses Following Timothy Grass Subcutaneous and Sublingual Immunotherapy,” Journal of Allergy and Clinical Immunology 148, no. 4 (2021): 1061–1071.e1011. 33819508 10.1016/j.jaci.2021.03.030 PMC12400438

[53] N. N. Bahceciler , C. Arikan , A. Taylor , et al., “Impact of Sublingual Immunotherapy on Specific Antibody Levels in Asthmatic Children Allergic to House Dust Mites,” International Archives of Allergy and Immunology 136, no. 3 (2005): 287–294. 15722639 10.1159/000083956

[54] C. J. Aranda , E. Gonzalez‐Kozlova , S. P. Saunders , et al., “IgG Memory B Cells Expressing IL4R and FCER2 Are Associated With Atopic Diseases,” Allergy 78, no. 3 (2023): 752–766. 36445014 10.1111/all.15601 PMC9991991

[55] E. Tissino , A. Gaglio , A. Nicolò , et al., “The VLA‐4 Integrin Is Constitutively Active in Circulating Chronic Lymphocytic Leukemia Cells via BCR Autonomous Signaling: A Novel Anchor‐Independent Mechanism Exploiting Soluble Blood‐Borne Ligands,” Leukemia 38, no. 10 (2024): 2127–2140. 39143370 10.1038/s41375-024-02376-7 PMC11436378

[56] C. Vogelberg , L. Klimek , B. Brüggenjürgen , and M. Jutel , “Real‐World Evidence for the Long‐Term Effect of Allergen Immunotherapy: Current Status on Database‐Derived European Studies,” Allergy 77, no. 12 (2022): 3584–3592. 36074052 10.1111/all.15506 PMC10087412

[57] M. E. Starrenburg , M. B. Imam , J. F. Lopez , et al., “Dupilumab Treatment Decreases MBC2s, Correlating With Reduced IgE Levels in Pediatric Atopic Dermatitis,” Journal of Allergy and Clinical Immunology 154, no. 5 (2024): 1333–1338. 39038586 10.1016/j.jaci.2024.06.023

[58] A. R. Dvorscek , J. Mulder , Z. Ding , M. C. van Zelm , D. M. Tarlinton , and M. J. Robinson , “IL‐21 Contributes to Type 2 Memory B Cell Formation,” Journal of Immunology 214, no. 8 (2025): 1898–1905. 10.1093/jimmun/vkaf147 40611513

[59] K. Bruton , A. Phelps , A. Ariaz , et al., “Pathogenic IgE‐Fated Memory B Cell Responses Retain Functional Plasticity,” Science Immunology 11, no. 115 (2026): eady2268. 41481696 10.1126/sciimmunol.ady2268

