# Supplementary material for: Biphasic Effects on Allergen‐Specific Type 2 Memory B Cells Over 18 Months Sublingual Immunotherapy for House Dust Mite Allergy
Source: Allergy. 2026 Apr 17;81(6):2172–83. doi: 10.1111/all.70342 (PMC13256270; doi:10.1111/all.70342)
Supplement: Supplementary file 1 — Figure S1: Allergen‐specific Bmem gating strategy. (A) From all events, the singlet live cells were gated, with subsequent gating of the lymphocyte population using CD45 and SSC. CD3+ T and CD19+ B‐cells were discriminated within lymphocytes, followed by the subsetting of B cells into CD38dim mature B cells, transitional B cells, and plasmablasts. Within the CD38dim cells, Bmem were defined by excluding naive B cells (CD27‐IgD+). Bmem were separated using IgM and IgD into unswitched Bmem (IgM+ only, IgD+ only, and IgM + IgD+) and Immunoglobulin (Ig) class‐switched populations. Ig‐switched subsets were then subsetted based on the differential expression of IgG1, IgG2, IgG3, IgG4, IgA, and IgE. (B) The detection of Der p 1 and (C) Der p 2 specific cells within Bmem and subsequent evaluation of Ig isotype and IgG subclass expression, as in panel A. (D) Within the total, Der p 1 and Der p 2 specific Bmem, the CD38dimCD21lo atypical Bmem population was defined. Figure S2: Der p 1 and Der p 2 tetramer staining and streptavidin‐only control double‐discrimination‐stained Bmem in the treatment group Representative plots from three participants on (A) Der p 1‐ and (B) Der p 2‐ tetramer staining compared to streptavidin‐only control at t = 0 and t = 18 of SLIT treatment. Figure S3: CD38dimCD21lo expressing‐ allergen‐specific Bmem over 18‐months in patients without or with SLIT. Frequencies of CD38dimCD21lo events within the (A) total, (B) Der p 1, and (C) Der p 2 Bmem. Individual data points are shown with median lines: Blue, no‐AIT; red, HDM‐SLIT. Statistics: The non‐parametric Friedman test and/or post hoc Dunn's multiple comparisons test; *p < 0.05. Figure S4: Numbers and immunophenotypes of allergen‐specific type 2 Bmem of the no‐AIT patient group. Bmem were defined within CD38dim B cells through the exclusion of naive B cells (CD27− IgD+; Fig. S1). Within (A) total, Der p 1‐ and Der p 2‐specific Bmem, IL4R+CD23+ type 2 Bmem were defined. Within type 2 Bmem, cells expressi [file ALL-81-2172-s001.docx]

**Supplementary materials** (4 Tables; 6 Figures)

Supplementary Table 1. Timing of sample collections

| **Timepoint**  **(months)** | **t=4** | **t=12** | **t=18** |
| --- | --- | --- | --- |
| **No-AIT** (n=17)  median days (range) | 133 (105-183) | 394 (337-413) | 587 (505-692) |
| **HDM-SLIT** (n=21)  median days (range) | 110 (93-121) | 359 (284-395) | 551 (470-603) |
| **p-value** | **<0.0001** | **0.0012** | **0.0846** |

Supplementary Table 2. List of antibodies used for Trucount analysis

| **Marker** | **Fluorochrome** | **Clone** | **Source** | **Cat. number** | **Volume per test**  **(µL/70µL)** | **Tube(s)** |
| --- | --- | --- | --- | --- | --- | --- |
| CD3 | FITC | UCHT1 | BD Biosciences | 555332 | 3 | 1 |
| CD4 | PC7 | SFCI12T4D11 | Beckman Coulter | 6607101 | 0.2 | 1 |
| CD8A | APC-H7 | SK1 | BD Biosciences | 560179 | 4 | 1 |
| CD16 | PE | B73.1 | Biolegend | 360704 | 0.5 | 1 |
| CD19 | APC | SJ25C1 | Biolegend | 363006 | 0.4 | 1 |
| CD45 | PerCP-Cy5.5 | 2D1 | BD Biosciences | 340953 | 5 | 1 |
| CD56 | PE | B159 | BD Biosciences | 555516 | 5 | 1 |

Supplementary Table 3. List of antibodies used in memory B cell flow cytometry

|  | **Fluorochrome** | **Clone** | **Source** | **Cat. number** | **Volume per test (µL/250 µL)** | **Tube(s)** |
| --- | --- | --- | --- | --- | --- | --- |
| CD3 | BUV805 | UCHT1 | BD Biosciences | 612895 | 6.25 | 2, 3 |
| CD19 | cFluor BYG710 | HIB19 | Cytek Biosciences | R7-20009 | 1.5 | 2, 3 |
| CD21 | BV711 | B-ly4 | BD Biosciences | 563163 | 1.25 | 2, 3 |
| CD23 | RealYellow610 | EBVCS-5 | BD Biosciences | 759529 | 2.5 | 2, 3 |
| CD27 | RB744 | O323 | BD Biosciences | 757041 | 6.25 | 2, 3 |
| CD29 | BUV563 | TS2/16 | BD Biosciences | 755227 | 1.56 | 2, 3 |
| CD38 | APC R700 | HIT2 | BD Biosciences | 564979 | 6.25 | 2, 3 |
| CD45 | cFluor V547 | HI30 | Cytek Biosciences | R7-20011 | 1.25 | 2, 3 |
| IL4Ra | RB613 | hIL4R-M57 | BD Biosciences | 759183 | 3 | 2, 3 |
| IgD | APC FIRE 750 | W18340F | BioLegend | 348238 | 1.25 | 2, 3 |
| IgM | Pacific Blue | MHM-88 | Biolegend | 314514 | 0.5 | 2, 3 |
| IgG1 | PE | G17-1 | BD Biosciences | 624049 (custom) | 0.25 | 2 |
| IgG2 | PE | SAG2 | Cytognos | CYT-IGG2PE | 5 | 2 |
| IgG2 | FITC | SAG2 | Cytognos | CYT-IGG2F | 5 | 2 |
| IgG3 | FITC | HP6047 | BD Biosciences | 624045 (custom) | 1.25 | 2 |
| IgG4 | APC | SAG4 | Cytognos | CYT-IGG4AP | 5 | 2 |
| IgA | PE-Vio770 | REA1014 | Miltenyi Biotec | 130-116-883 | 2.5 | 2 |
| IgE | BV786 | G7-26 | BD Biosciences | 744320 | 1.56 | 2 |
| Streptavidin | BV421 | - | Biolegend | 405225 | 2.0 | 2, 3 |
| Streptavidin | BV480 | - | BD Biosciences | 564876 | 8.7 | 2, 3 |
| Streptavidin | BUV395 | - | BD Biosciences | 564176 | 5.7 | 2, 3 |
| Streptavidin | BUV737 | - | BD Biosciences | 564293 | 8.7 | 2, 3 |
| Viability dye | Live Dead Blue | - | Life Technologies | L23105 | 0.25 | 2, 3 |

Supplementary Table 4. Composition of the antibody panels

| **Fluoro**  **-chrome** | **BUV**  **395** | **Live Dead Blue** | **BUV**  **563** | **BUV**  **737** | **BUV**  **805** | **BV**  **421** | **Pacific Blue** | **BV**  **480** | **cFluor V547** | **BV**  **711** | **BV**  **786** | **FITC** | **RB**  **613** | **PerCP-Cy5.5** | **RB**  **744** | **PE** | **RealYellow**  **610** | **cFluor BYG**  **710** | **PC7/ PE-Vio**  **770** | **APC** | **APC R700** | **APC-H7/ APC FIRE 750** |
| --- | --- | --- | --- | --- | --- | --- | --- | --- | --- | --- | --- | --- | --- | --- | --- | --- | --- | --- | --- | --- | --- | --- |
| **1. Trucount** | - | - |  | - | - | - | - | - | - | - | - | CD3 | - | CD45 |  | CD16 +  CD56 |  |  | CD4 | CD19 | - | CD8A |
| **2. Ag-specific Bmem** | [Der p 1]_4_-strep | Viability | CD29 | [Der p 2]_4_-strep | CD3 | [Der p 1]_4_-strep | IgM | [Der p 2]_4_-strep | CD45 | CD21 | IgE | IgG2/ IgG3 | CD124 (IL4Rα) | - | CD27 | IgG1/IgG2 | CD23 | CD19 | IgA | IgG4 | CD38 | IgD |
| **3. Streptavidin control** | Strep | Viability | CD29 | Strep | CD3 | Strep | IgM | Strep | CD45 | CD21 | - | - | CD124 (IL4Rα) | - | CD27 | - | CD23 | CD19 | - | - | CD38 | IgD |


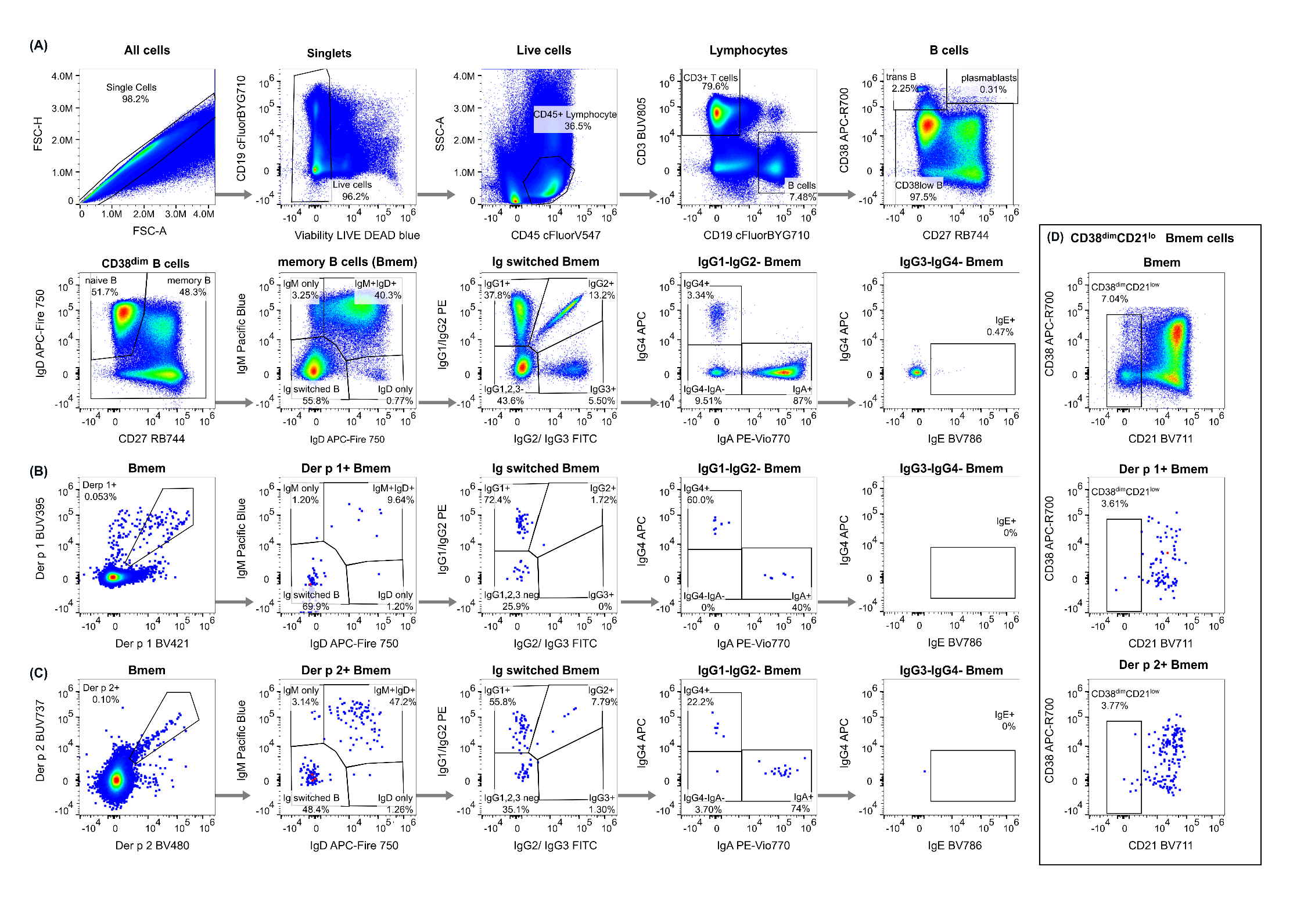
 **Supplementary Figure** 1. Allergen-specific Bmem gating strategy. (**A**) From all events, the singlet live cells were gated, with subsequent gating of the lymphocyte population using CD45 and SSC. CD3^+^ T and CD19^+^ B-cells were discriminated within lymphocytes, followed by the subsetting of B cells into CD38^dim^ mature B cells, transitional B cells and plasmablasts. Within the CD38^dim^ cells, Bmem were defined by excluding naive B cells (CD27-IgD+). Bmem were separated using IgM and IgD into unswitched Bmem (IgM+ only, IgD+ only and IgM+IgD+) and Immunoglobulin (Ig) class-switched populations. Ig-switched subsets were then subsetted based on the differential expression of IgG1, IgG2, IgG3, IgG4, IgA and IgE. (**B**) The detection of Der p 1 and (**C**) Der p 2 specific cells within Bmem and subsequent evaluation of Ig isotype and IgG subclass expression as in panel A. (**D**) Within the total, Der p 1 and Der p 2 specific Bmem, the CD38^dim^CD21^lo^ atypical Bmem population was defined.


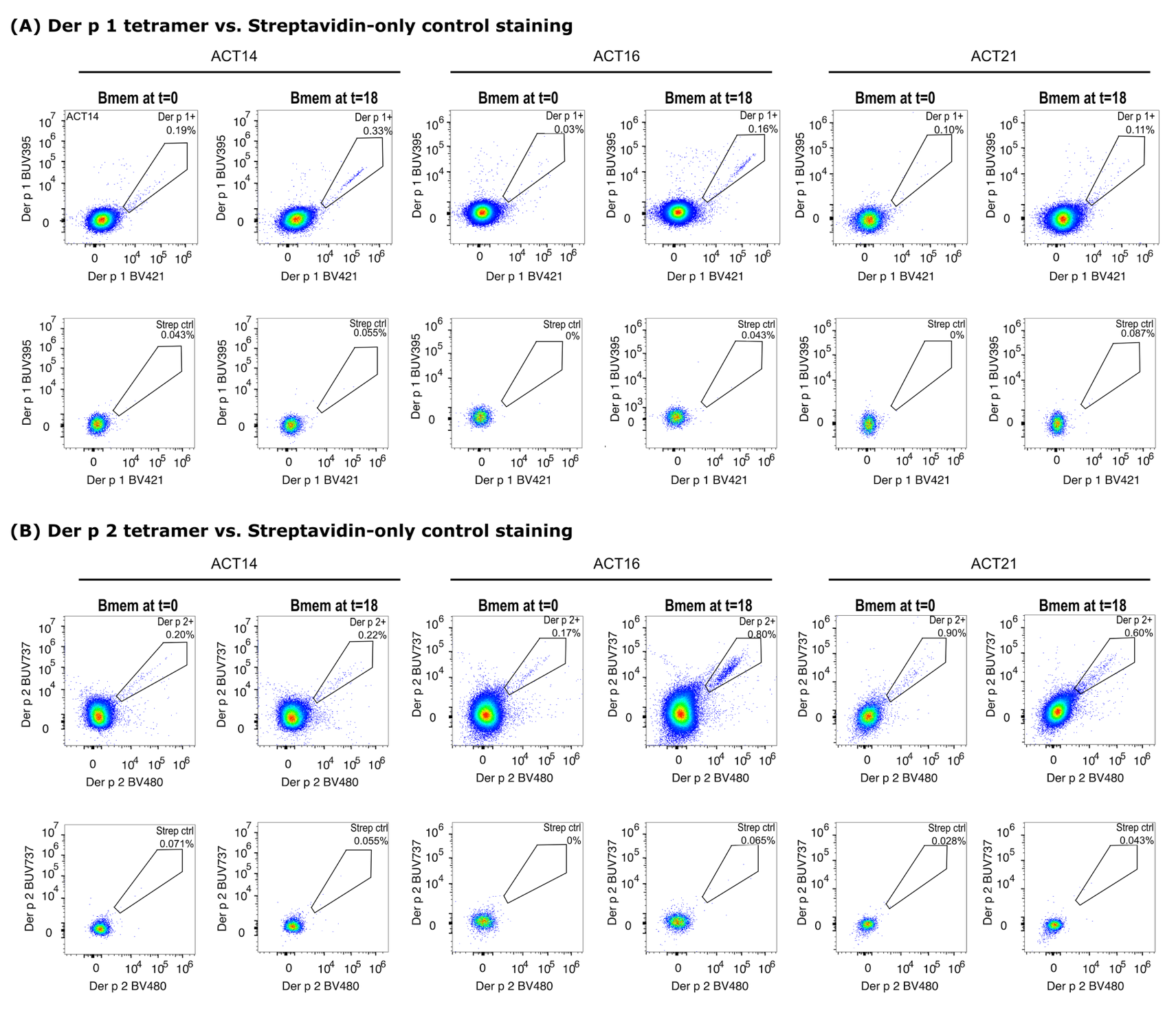


Supplementary Figure 2. Der p 1 and Der p 2 tetramer staining and streptavidin-only control double-discrimination-stained Bmem in treatment group. Representative plots from three participants on (**A**) Der p 1- and (**B**) Der p 2- tetramer staining compared to streptavidin-only control at t=0 and t=18 of SLIT treatment.


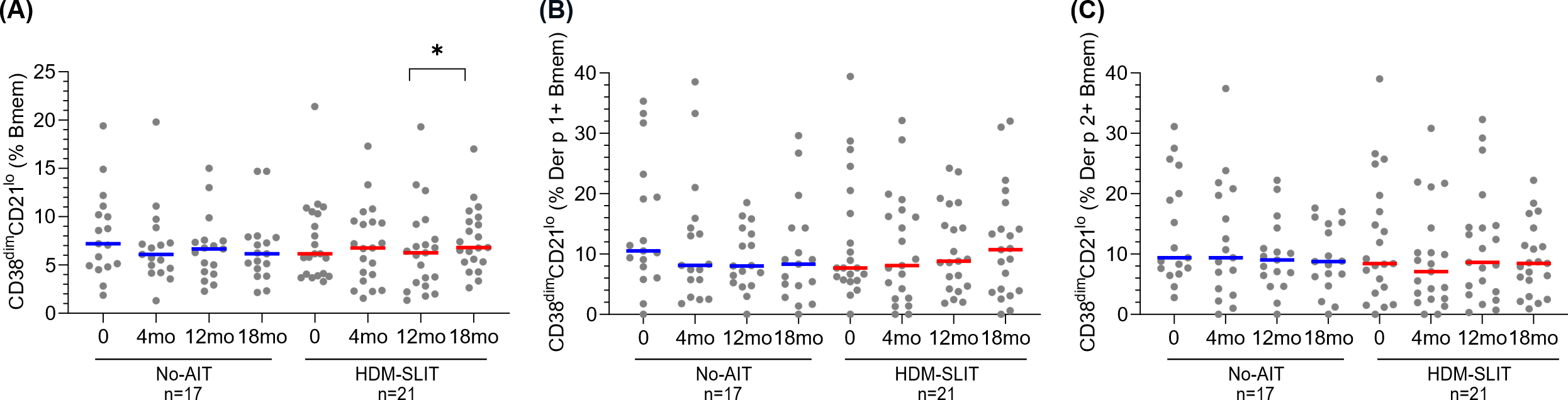


Supplementary Figure 3. CD38^dim^CD21^lo^ expressing- allergen-specific Bmem over 18-months in patients without or with SLIT. Frequencies of CD38^dim^CD21^lo^ events within the (**A**) total, (**B**) Der p 1 and (**C**) Der p 2 Bmem. Individual data points are shown with median lines; blue, no-AIT; red, HDM-SLIT. Statistics: The non-parametric Friedman test and/or post hoc Dunn’s multiple comparisons test; * p < 0.05.


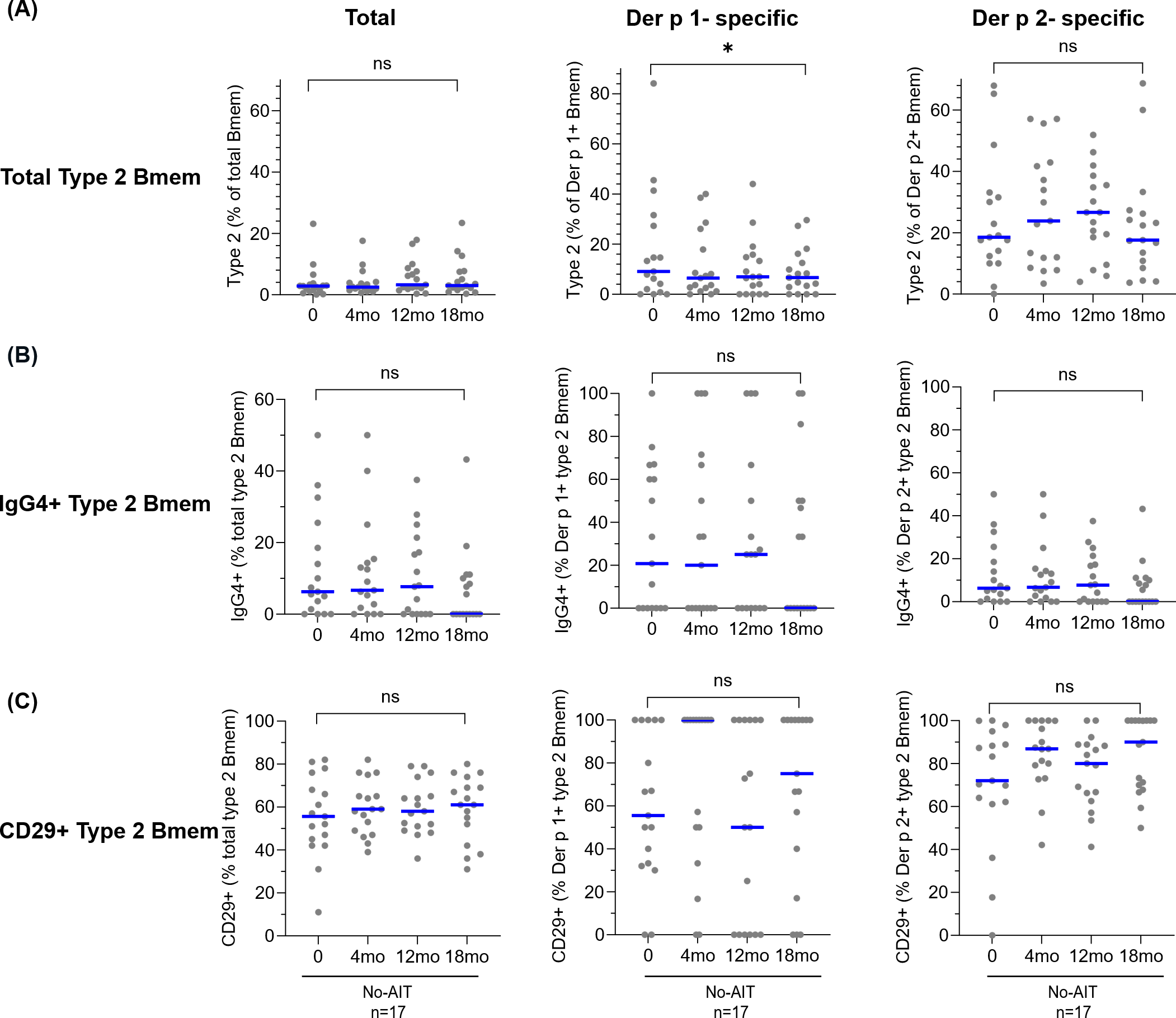


Supplementary Figure 4. Numbers and immunophenotypes of allergen-specific type 2 Bmem of the no-AIT patient group. Bmem were defined within CD38^dim^ B cells through the exclusion of naive B cells (CD27^-^ IgD^+^; **Fig. S1**). Within (**A**) total, Der p 1- and Der p 2-specific Bmem, IL4R^+^CD23^+^ type 2 Bmem were defined. Within type 2 Bmem, cells expressing (**B**) IgG4 and (**C**) CD29 were defined. Single data points are shown with blue lines denoting median values. Statistics: Normality Shapiro-Wilk tests. Data followed non-Gaussian distribution, the non-parametric Friedman test and/or post hoc Wilcoxon matched-pairs signed-rank test was used; *p < 0.05; ns, not significant.

**
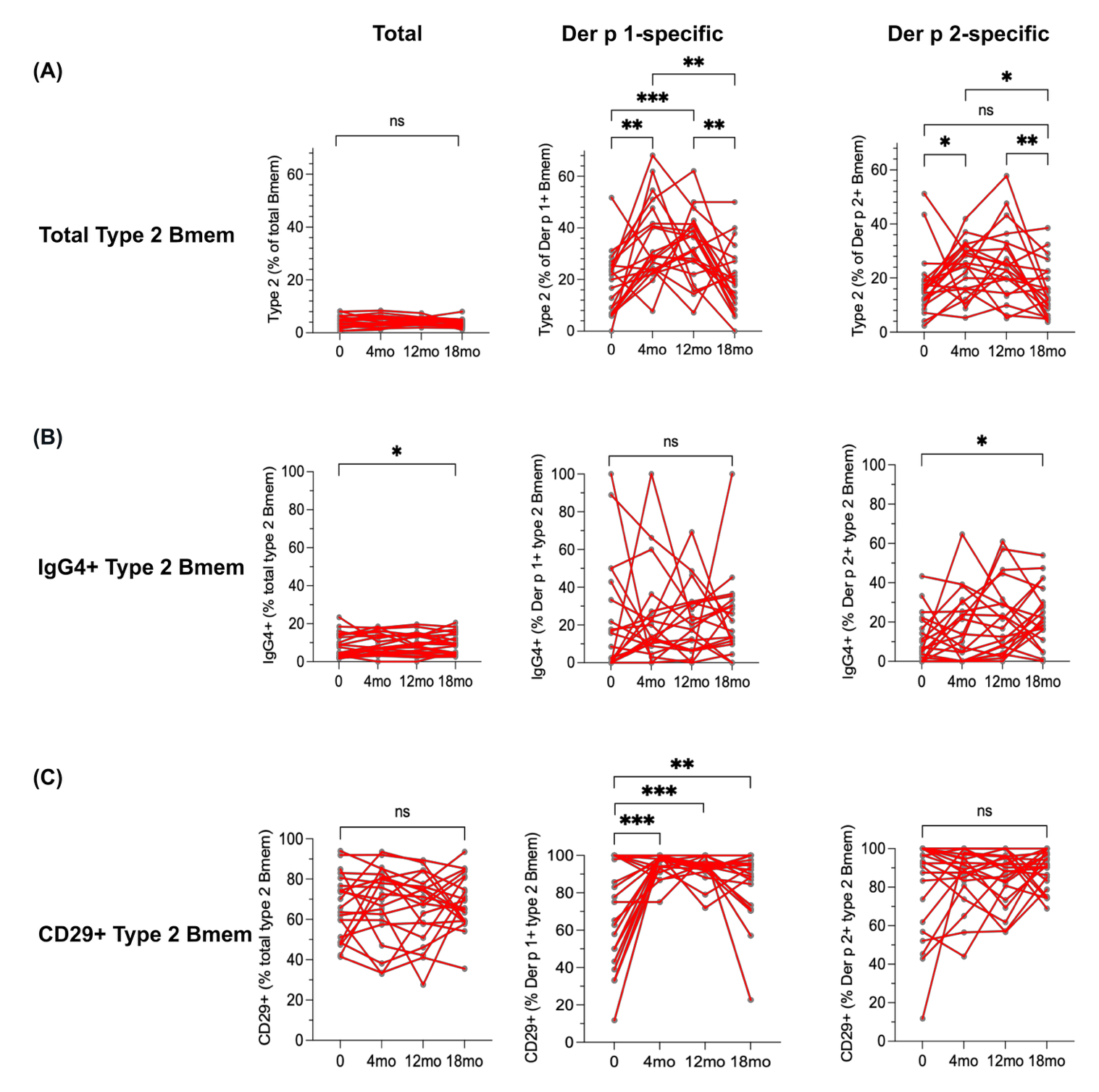
**Supplementary Figure 5. Kinetics of the frequency of the total and allergen-specific Type 2 Bmem and the subsets expressing IgG4+ and CD29+ across 18 months of treatment. (**A**) The kinetics of the frequencies of the total, Der.p 1 and Der p 2 (**A**) Type 2 Bmem (**B**) IgG4-expressing Type 2 Bmem, and (**C**) CD29-expressing Type 2 Bmem of across 18 months of treatment in HDM-SLIT subjects. Statistics: The non-parametric Friedman test and/or post hoc Wilcoxon matched-pairs signed-rank test w; *p < 0.05; **p < 0.01; ***p < 0.001; ns, not significant.


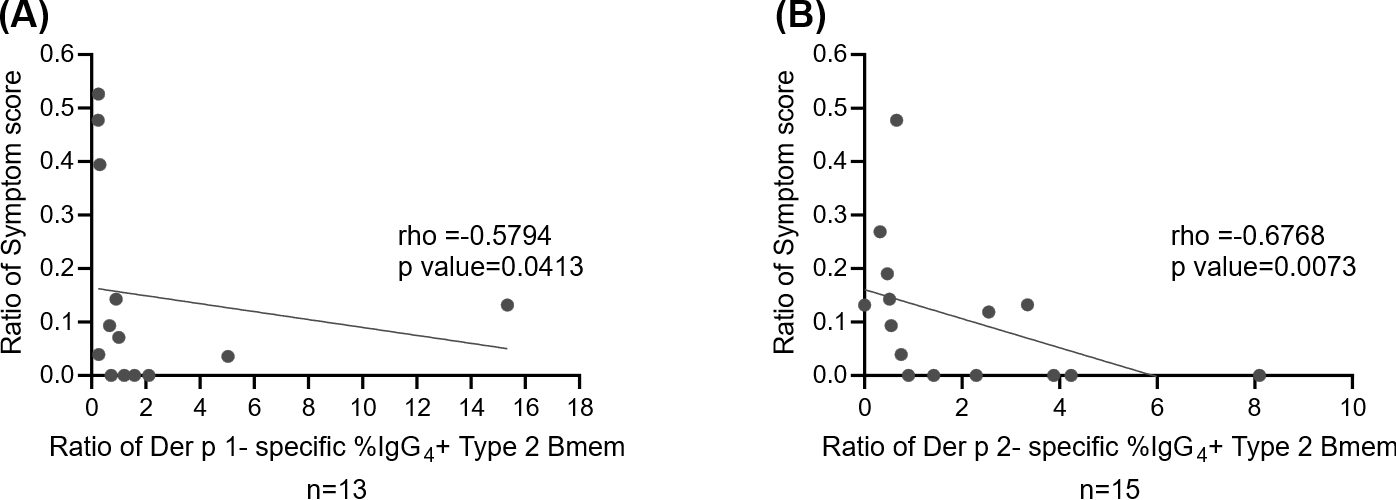


Supplementary Figure 6. Correlation analysis of the change in clinical symptoms with the change in allergen-specific IgG4+ Type 2 Bmem. Ratios of the frequencies of IgG4^+^ events 18 months over 0 months within (**A**) Der p 1- and **(B)** Der p 2-specific Type 2 Bmem (IL4Rα^+^ CD23^+^) plotted versus the ratio of the symptom scores at 18 over 0 months of treatment. Individual data points are shown with linear regression lines. Sample sizes for each measurement are indicated, where samples with zero event of specific Type 2 Bmem were excluded from analysis. Spearman's rank correlation tests were conducted, with the rho correlation coefficient indicating the monotonic relationship between the subset and clinical parameter, and the p-values the significance of the correlation tests.
